# Functional human iPSC-derived alveolar-like cells cultured in a miniaturized 96‑Transwell air–liquid interface model

**DOI:** 10.1038/s41598-021-96565-4

**Published:** 2021-08-23

**Authors:** Teresa Bluhmki, Stefanie Traub, Ann-Kathrin Müller, Sarah Bitzer, Eva Schruf, Marie-Therese Bammert, Marcel Leist, Florian Gantner, James P Garnett, Ralf Heilker

**Affiliations:** 1 Department of Drug Discovery Sciences, Boehringer Ingelheim Pharma GmbH & Co. KG, 88397 Biberach an der Riss, Germany; 2 Trenzyme GmbH, Byk-Gulden–Str. 2, 78467 Constance, Germany; 3 Department of Immunology & Respiratory Diseases Research, Boehringer Ingelheim Pharma GmbH & Co. KG, 88397 Biberach an der Riss, Germany; 4 In-vitro Toxicology and Biomedicine, University of Konstanz, 78457 Constance, Germany; 5 Department of Translational Medicine and Clinical Pharmacology, C. H. Boehringer Sohn AG & Co. KG, 88397 Biberach an der Riss, Germany

**Keywords:** Biochemistry, Cell biology, Drug discovery, Molecular biology, Stem cells, Biomarkers, Diseases

## Abstract

In order to circumvent the limited access and donor variability of human primary alveolar cells, directed differentiation of human pluripotent stem cells (hiPSCs) into alveolar-like cells, provides a promising tool for respiratory disease modeling and drug discovery assays. In this work, a unique, miniaturized 96-Transwell microplate system is described where hiPSC-derived alveolar-like cells were cultured at an air–liquid interface (ALI). To this end, hiPSCs were differentiated into lung epithelial progenitor cells (LPCs) and subsequently matured into a functional alveolar type 2 (AT2)-like epithelium with monolayer-like morphology. AT2-like cells cultured at the physiological ALI conditions displayed characteristics of AT2 cells with classical alveolar surfactant protein expressions and lamellar-body like structures. The integrity of the epithelial barriers between the AT2-like cells was confirmed by applying a custom-made device for 96-parallelized transepithelial electric resistance (TEER) measurements. In order to generate an IPF disease-like phenotype in vitro, the functional AT2-like cells were stimulated with cytokines and growth factors present in the alveolar tissue of IPF patients. The cytokines stimulated the secretion of pro-fibrotic biomarker proteins both on the mRNA (messenger ribonucleic acid) and protein level. Thus, the hiPSC-derived and cellular model system enables the recapitulation of certain IPF hallmarks, while paving the route towards a miniaturized medium throughput approach of pharmaceutical drug discovery.

## Introduction

Interstitial lung diseases (ILDs) incorporate a large group of parenchymal lung disorders affecting the pulmonary interstitium, which cause progressive scarring and inflammation of the lung tissue. Risk factors may include smoking, environmental exposures and family history of the disease^1^, but a significant proportion of cases is rated as idiopathic, implying an unknown cause of the disease^1^. Idiopathic pulmonary fibrosis (IPF) is the most common and pernicious type among ILDs with unknown etiology. It is characterized through progressive fibrosis within the lung epithelium^1–3^, involving alveolar epithelial cells but also macrophages, monocytes and local fibroblasts^4^. Current treatment options are highly limited^3^, no other drugs than nintedanib (Ofev) and pirfenidone (Esbriet) are approved for the treatment of IPF^5^. For both drugs, cellular effects on the level of fibroblasts and anti-inflammatory activities have been described^3^. In contrast, disease intervention on the level of the epithelial cells has so far not been thoroughly explored^6^.

A chronic damage of the alveolar epithelium is thought to be causal in the pathogenesis of IPF^7^. The human alveolar epithelium consists of alveolar type 1 (AT1) and type 2 (AT2) cells. AT2s exhibit a cuboidal morphology and cover 3–5% of the alveoli surface area^8^. They play key roles in the maintenance of a proper alveolus function possessing different protective and regenerative properties^9–11^. They carry out the synthesis, secretion and recycling of different substances like pulmonary surfactants including surfactant protein A, B, C, and D (SFTPA, SFTPB, SFTPC, SFTPD) and other protective biomolecules^1,8^. With the aid of these surfactants, proper surface tension can be maintained stabilizing alveolar size, keeping the alveoli dry and facilitating breathing^12^. Besides, the AT2 cells are not only capable of proliferating into new AT2 cells, but they also serve as facultative progenitors for AT1 cells, thus being involved in alveolar repair and homeostasis^13,14^. The specialized AT1 cells take up a large portion of the total surface of the human alveoli and serve as key players in the regulation of the gas exchange^9,15^. With their flattened and branched morphology, they build a very large and thin apical surface, which enables them to conduct a potent gas exchange^16^. Importantly, both alveolar cells types are closely connected through tight-junctions (TJs) to form a robust epithelial barrier. Dysfunctional AT2 cells are presumably linked to fibrotic processes in the lung^17,18^, including chronic obstructive pulmonary disease^2^, pulmonary fibrosis^4,5^ and lung cancer^19^. To foster drug discovery in this regard, a translational cellular in vitro model of the alveolar epithelial layer is urgently needed to support pre-clinical research projects addressing causes and treatment of IPF in the human lung.

To better understand the various mechanisms driving the disease, it is important to have a predictive in vitro system that can resemble the human physiological situation. Immortalized cell lines, such as lung adenocarcinoma (A549) cells, have been widely used instead of primary cells^20,21^. However, they fail to reproduce several features of the physiological epithelium due to their phenotypes altered by culture conditions^22,23^. Models based on human primary small airway and alveolar epithelial cells are currently the most predictive ones, but they have also their limitations. Despite the well-known proliferative capacity of AT2 cells in vivo, however, primary AT2 cells in vitro may not be expanded beyond a few passages^24,25^. Importantly, the use of primary alveolar cells is further complicated by their tendency to spontaneously trans-differentiate to terminally differentiated AT1 cells^26^. Additionally, the access to non-diseased human primary alveolar cells is highly limited and cost intensive, rendering them less suitable for the application in large-scale cultures and medium throughput screenings^27^.

An alternative strategy is working with human induced pluripotent stem cells (hiPSCs). Since Takahashi and Yamanaka established the nuclear reprogramming of adult cells to a pluripotent cell fate in 2006^28^, these cells have been explored as an alternative cell source for the replacement of cell lines and primary cells. This technology offers an unlimited number of tissue-specific cells that can be used in highly predictive in vitro models^29^. Ideally, hiPSC-derived cell populations would include the different subtypes of alveolar epithelial cells, which would resemble the physiological situation of the human lung^30,31^. Such a controlled and targeted differentiation of hiPSCs towards alveolar cells would be an ideal cellular model for studying epithelial dysfunction in IPF. However, the differentiation of hiPSCs to lung epithelial cells has been challenging: While various differentiation protocols are available in literature^32,33^, so far, the hiPSC-derived alveolar cells have not thoroughly been studied under ALI conditions^34^. Recent studies reported protocols for the directed generation of functional stem cell-derived alveolar cells and their applications in respiratory disease research^32,35–39^. These latter studies relied on directed in vitro differentiation of hiPSCs into alveolar cells based on the induction of Definitive Endoderm (DE)^40^, followed by Anterior Foregut Endoderm (AFE) specification, patterning into ventral Anterior Foregut (vAFE) fate, into lung progenitor cells (LPCs) and subsequent maturation into various lung epithelial cell types^33,41,42^. One key step was the development of NK2 Homeobox 1 (NKX2-1^+^) LPCs using a mixture of growth factors, that can be subsequently matured into an alveolar fate by continued culture under submerged or 3D organoid conditions^35,43,44^. In contrast, other protocols position the in vitro epithelium on a synthetic membrane at the boundary between air and culture medium, known as air–liquid interface (ALI)^45–47^. However, most of the published protocols contain 3D differentiation steps, such as embryoid body or organoid formation. Additionally, they are restricted to the commonly used large scale formats (e.g. 24-Transwell plates) which hamper the application of ALI conditions to higher throughput profiling of drug candidates. A 2D monolayer-like differentiation protocol combined with a cell banking step of LPCs has never been reported yet. In this work, the flask-based LPC generation was decoupled from the 96-Transwell-based final maturation into AT2-like cells. Although, hiPSC-derived lung epithelial cell models have been used for disease modelling^45,48^, they have so far never been miniaturized and applied to medium throughput applications to a similar extent.

In summary, the aim of the work was to establish a stepwise differentiation protocol of hiPSCs into human alveolar-like cells, including cell banking steps of intermediate stages, for the study of epithelial dysfunction in IPF. In addition, a miniaturized 96-Transwell ALI format was developed, opening new routes towards target-focused and phenotypic drug discovery^49^.

## Results

### Generation of functional monolayer AT2-like cells out of hiPSCs including cell banking steps of intermediate stages

**Figure 1:**
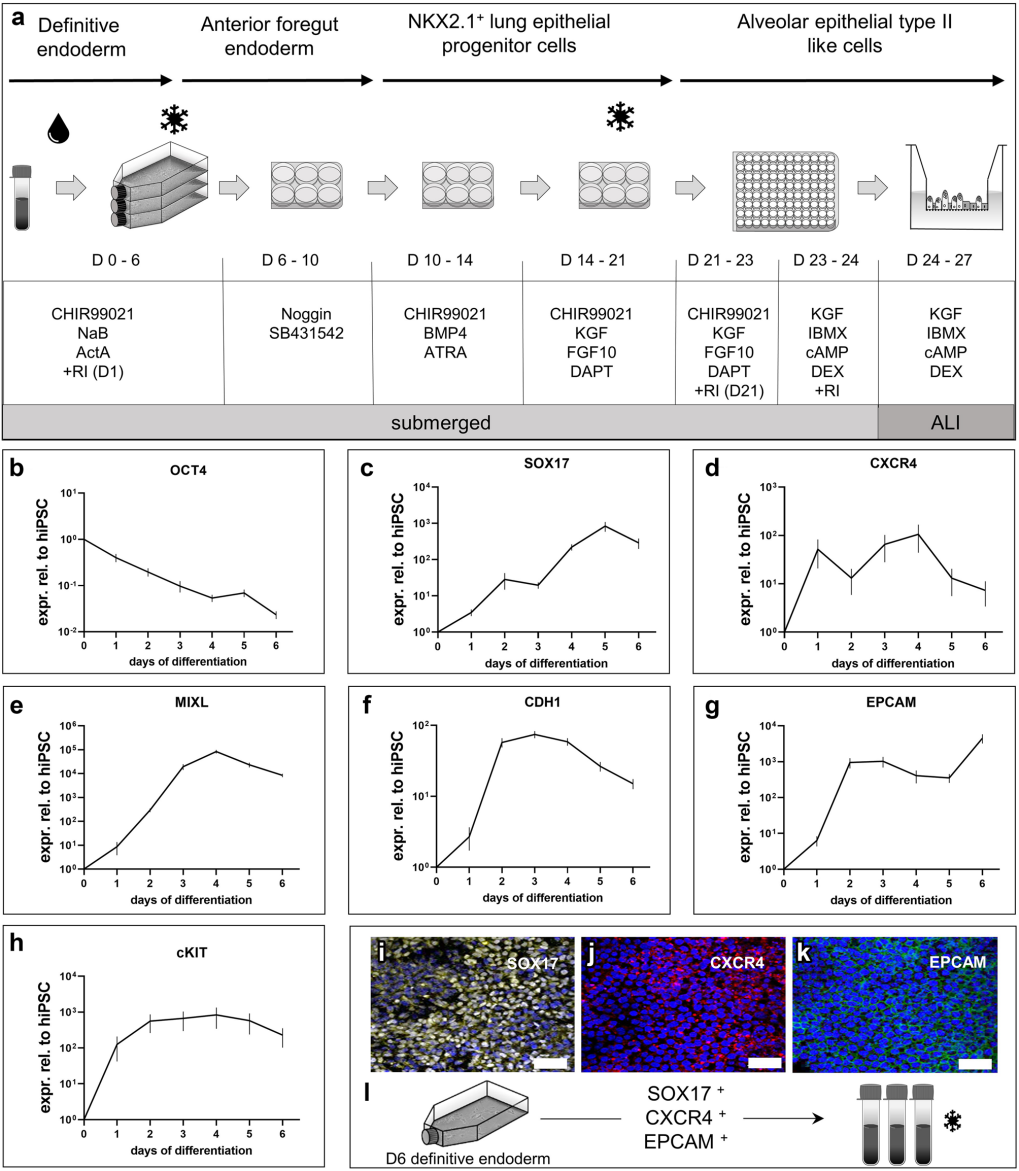
Optimized stepwise differentiation protocol of induced pluripotent stem cells towards alveolar-like cells including cell banking steps. (**a**) Schematic of the stepwise differentiation triggered through different proteins and small molecules. NaB = Sodium Butyrate, ActA = Activin A, RI = Y27632, ATRA = All-trans-retinoic-acid, DEX = dexamethasone. (**b**–**h**) Relative expression levels at daily intervals during differentiation to definitive endoderm (DE) confirms up-regulation of DE markers within 6 days. Targeted differentiation leads to loss of pluripotency within 6 days. (**i**–**k**) Representative immunofluorescence staining for classical DE marker expression in hiPSCs following 6 days of directed differentiation (**i**: SOX17, **j**: CXCR4, **k**: EpCAM, nuclei: Hochest33342). Scale bar = 50 µm. (**l**) Schematic of included freezing step at day 6 of DE differentiation.

Within this work the generation of functional hiPSC-derived AT2-like cells was successfully achieved through the directed in vitro differentiation of hiPSCs involving the recapitulation of the complex in vivo development of the lung. This was accomplished through precise timing of activation or inhibition of specific signaling pathways relevant in the embryogenesis of the human lung. LPC maturation toward AT2-like cells was carried out on 96-Transwell inserts at ALI to resemble the physiological environment of the mature human alveolar epithelium. The here described differentiation protocol (illustrated in Fig. 1a), which focused on culture conditions and suitability in upscaled applications, incorporates various elements of previously established methods, e.g. of Gotoh et al.^35^ and Yamamoto et al.^50^. This protocol allows for the efficient generation of functional 2D AT2-like cells out of hiPSCs including cell banking steps of intermediate stages.

First, the selected ChiPSC18 line was characterized with respect to pluripotency. Both on protein and mRNA (messenger ribonucleic acid) level, the selected hiPSCs expressed classical pluripotency markers, such as octamer-binding transcription factor 4 (*OCT4*), Nanog Homeobox (*NANOG*) and SRY-Box Transcription Factor 2 (*SOX2*) (see Supplementary Fig. S1a–e, online). Additionally, the differentiation potential towards all three germ layers was confirmed by immunofluorescence staining of SRY-Box Transcription Factor 17 (SOX17)/C-X-C Motif Chemokine Receptor 4 (CXCR4) (Endoderm), Nestin (Ectoderm) and Brachyury (Mesoderm) (see Supplementary Fig. S1f–i, online). By subsequently applying a previously established scalable DE expansion protocol^40^, the generated DE cells displayed key markers such as *SOX17, CXCR4*, Mix Paired-Like Homeobox (*MIXL*), E-Cadherin (*CDH1*), Epithelial Cell Adhesion Molecule (*EPCAM*), KIT Proto-Oncogene and Receptor Tyrosine Kinase (*cKIT*) in a RT-PCR analysis. In parallel, the loss of pluripotency markers such as *OCT4* within 6 days of differentiation was demonstrated (Fig. 1b–h). DE cell identity was also confirmed by immunofluorescence staining for SOX17, CXCR4 and EPCAM. Before proceeding to the next steps of the differentiation protocol, the DE cells were cryopreserved and stored in aliquots (Fig. 1i–l).

**Figure 2:**
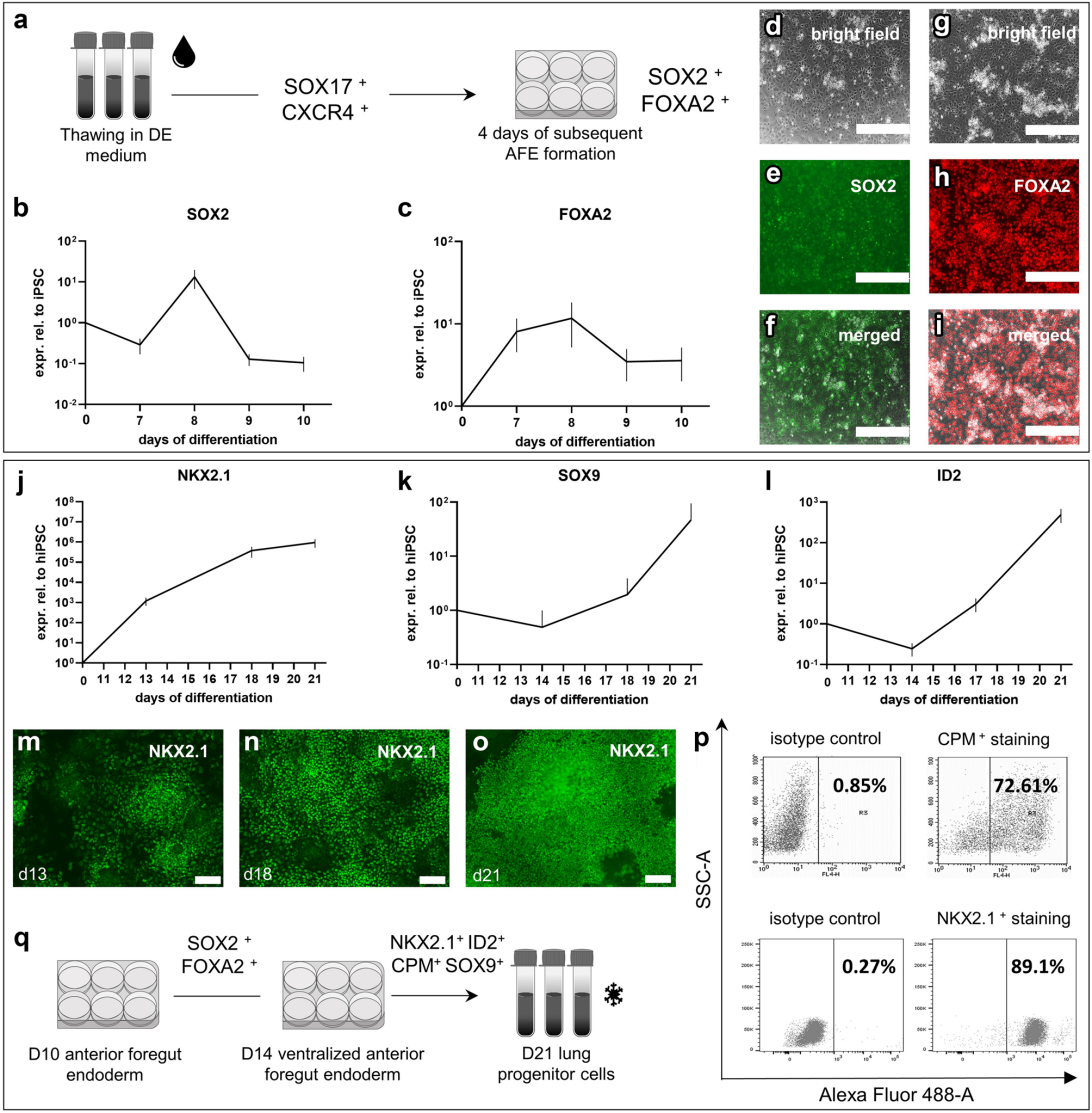
Generation of Lung Progenitor Cells (LPCs) following the Definitive Endoderm Formation. (**a**) Schematic of freezing thawing cycle and subsequent AFE differentiation of previous generated DE cells. (**b**,**c**) Relative expression levels during differentiation towards Anterior Foregut Endoderm (AFE) confirmed by the up regulation of the markers SOX2 and FOXA2 within following 4 days. (**d**–**i**) Representative immunofluorescence staining for AFE marker expression in hiPSCs following 4 days of directed differentiation (**d**: bright field, **e**: SOX2, **f**: merged bright field and SOX2; **g**: bright field, **h**: FOXA2, **i**: merged bright field and FOXA2). Scale bar = 400 µm. (**j**–**l**) Up-regulation of LPC markers NKX2.1, SOX9 and ID2 based on the efficient generation of ventralized AFE (vAFE) cells, within 10 days. (**m**–**o**) Representative immunofluorescence staining of the LPC marker NKX2.1 at day 13 (m), day 18 (**n**) and day 21 (**o**) of differentiation. Scale bar = 100 µm. (**p**) Representative FACS plots based on expression of CPM and NKX2.1 (marker for LPCs) in hiPSC-derived LPCs (day 21). Percentages of positive cells are shown in each plot based on isotype control gating strategies. (**q**) Schematic of differentiation process and essential marker expressions prior to lung progenitor cell banking. Depicted are means ± 95% CI of at least three independent experiments.

Frozen stocks of generated DE cells were used for the subsequent AFE transition (Fig. 2a). The differentiation potential was maintained during the cryopreservation step, indicated by the thawed cells expressing classical DE marker proteins and their typical polygonal and cuboidal morphology (see Supplementary Fig. S2a–c, online). A kinetics experiment of AFE formation demonstrated highest gene expression on day 8 of differentiation, verified by mRNA expression of AFE stage markers *FOXA2* (Forkhead Box A2) and *SOX2* (Fig. 2b,c). Corresponding protein expression was confirmed by immunofluorescence (Fig. 2d–i). In direct comparison, the cryopreserved DE stocks showed a differentiation potential towards AFE cells comparable to non-cryopreserved DE cells (see Supplementary Fig. S2d–i, online).

Following the patterning to vAFE and LPC induction, hiPSC-derived intermediate states displayed increased expression of *NKX2.1*, SRY-Box Transcription Factor 9 (*SOX9*) and Inhibitor of DNA Binding 2 (*ID2*) over time, with highest gene expression levels on day 21 of differentiation (Fig. 2j–l). These findings could be confirmed by the time-dependent protein expression of NKX2.1 detected by immunofluorescence staining (Fig. 2m–o). To ensure efficient generation of LPCs prior to cryopreservation, cells were analyzed regarding the lung progenitor markers Carboxypeptidase M (CPM) and NKX2.1. Based on flow cytometry data, 73% of the differentiated LPCs at day 21 were CPM positive and 89% NKX2.1 positive compared to the respective isotype controls (Fig. 2p). Kinetics of CPM protein expression in LPCs displayed highest expression at day 21, based on immunofluorescence staining (see Supplementary Fig. S3, online). Furthermore, unmeant patterning of LPCs towards thyroid like cells was excluded by the negative staining of Paired-Box-Protein 8 (PAX8) (0.14%) (Supplementary Fig. S13, online). A 3D LPC culture was utilized to additionally confirm the budding and branching potential of the hiPSC-derived LPCs, which constitutes a key functional feature of fetal epithelial progenitors during lung morphogenesis (see Supplementary Fig. S2j,k, online). Moreover, throughout the differentiation from hiPSCs (day 0) to LPCs (day 21), the number of cells increased by a factor of 4.8 (see Supplementary Fig. 2l, online). Based on these data sets cryopreservation and cell banking of LPCs was established on day 21 of hiPSC differentiation (Fig. 2q).

**Figure 3:**
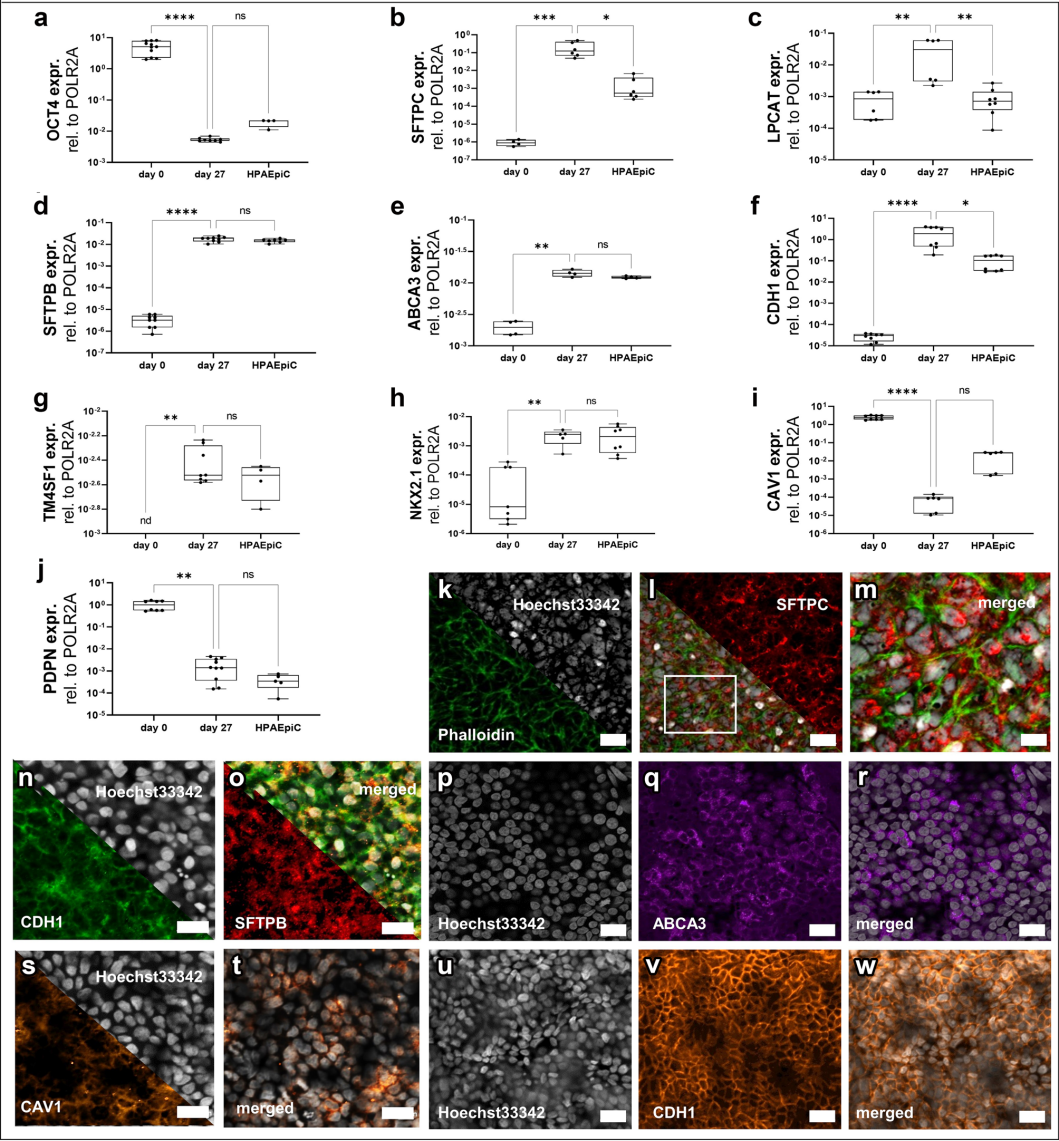
Confirmation of AT2-like phenotype of hiPSC-derived air–liquid interface (ALI) cultures verified on both protein and mRNA level. (**a**–**l**) One-step RT-PCR analysis of different lung cell markers. Statistical comparison between day 27 differentiated alveolar like cell, hiPSC (day 0) and HPAEpiC isolated total RNA. (**a**) OCT4 pluripotency marker (**b**–**e**) expression levels of classical AT2 cell markers. (**f**) CDH1 as a marker for epithelial cells. (**g**,**h**) TM4SF1 and NKX2.1 as alveolar lung progenitor marker. (**i**,**j**) Classical markers for alveolar type 1 cells. Median; range [min, max], N = 3; nd = not detectable, ns = not significant, *p < 0.05, **p < 0.01, ****p < 0.0001. (**k**–**m**) Immunofluorescence staining of SFTPC at day 27 of differentiation. (**k**) white: Hoechst33342, green: Phalloidin; (**l**) red: SFTPC, merged picture; (**m**) magnification of overlay. (**n**,**o**) SFTPB (red) and CDH1 (green) staining in AT2-like cells. Scale bar = 20 µm. (**p**–**r**) ABCA3 and (u-w) CDH1 staining in AT2-like cells after 27 days of differentiation. Scale bar = 20 µm. (**s**,**t**) Immunofluorescence staining of AT1 marker CAV1 (orange). Scale bar = 20 µm.

Subsequently, to induce specification into AT2-like cells, LPCs were seeded from cryopreserved cell stocks onto 96-Transwell inserts and confluent cultures were further differentiated at an air–liquid-interface until day 27. Finally, cells expressed mature AT2 markers such as *SFTPC*, *SFTPB* and ATP-binding cassette sub-family A member 3 (*ABCA3*) significantly higher compared to hiPSCs (day 0) (p < 0.0003, p < 0.0001, p < 0.0033) in line with the significant decrease of a pluripotency fate (*OCT4*, p < 0.0001). In addition, the expression of the lamellar body-associated protein *LPCAT-1* (Lysophosphatidylcholine acyltransferase 1) was also significantly induced during the differentiation process (p < 0.0029) (Fig. 3a–e). As shown in Fig. 3i,j, markers related to an AT1 phenotype such as *PDPN* (Podoplanin) and *CAV1* (Caveolin-1), were also present in the AT2-like cultures, whereas no significant differences in expression levels were detected compared to Human Pulmonary Alveolar Epithelial Cell (HPAEpiC) levels (p < 0.2258, p < 0.0790). Furthermore, differentiated cells on day 27 showed significantly upregulated expression of the human alveolar epithelial progenitor marker *TM4SF1* (member of the transmembrane 4 superfamily) (p < 0.0078), as well as the early lung progenitor marker *NKX2.1* compared to hiPSC (p < 0.0039) (Fig. 3g,h). Among other alveolar epithelial associated transcripts (see Supplementary Fig. S5a,b,g, online), the pan-epithelial marker *CDH1* as well as tight junction markers were highly expressed in the day 27 differentiated AT2-like cultures (Fig. 3f, Supplementary Fig. 5c–e, online). Comparison of protein analysis of surfactants in both hiPSC-derived AT2-like cells and HAEpiC showed a 2.4-fold higher protein expression for SFTPC and 1.7-fold higher expression of SFTPB (see Supplementary Fig. S4k, online). For statistical details see Supplementary Tables S4–15, online.

Immunofluorescence stainings showed the homogeneous expression of E-Cadherin (CDH1) and confirmed the presence of SFTPC^+^, ABCA3^+^, SFTPB^+^, CAV1^+^ cells, indicative of an AT2 cell phenotype (Fig. 3k–w). Semi-quantitative analysis of stainings revealed 87% ± 8.4 of cells were SFTPC^+^. Additionally, the hiPSC-derived AT2-like cells contained large organelles displaying intensive accumulation of SFTPC and also stain double positive for NKX2.1 and SFTPC, shown in Supplementary Figs. 4e–i; 4l–o, online. Furthermore, Hematoxylin and Eosin staining of cross-sectioned 96-Transwell cultures revealed physiological monolayer-like arrangement of AT2-like cells (see Supplementary Fig. 5h,i, online).

**Figure 4:**
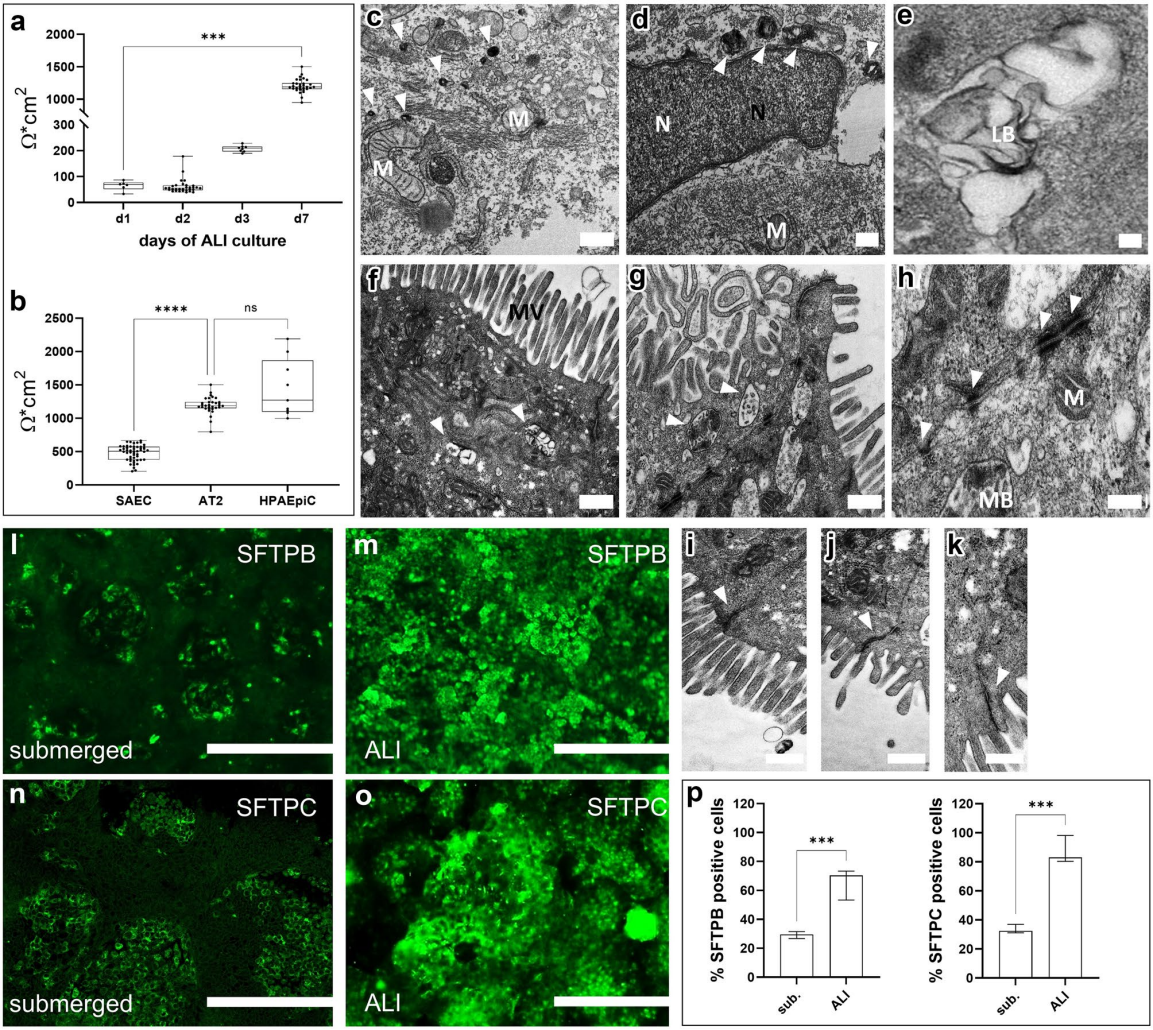
Characterization of hiPSC-derived alveolar like cells cultured under ALI conditions. (**a**) Change in TEER over 1 week of ALI culture. (**b**) Comparison of TEER values between primary human small airway cells (hSAEC), literature based human pulmonary alveolar epithelial cells (HPAEpiC) and the generated AT2-like cells. Median; range [min, max], N = 5; ns = not significant, ***p < 0.001, ****p < 0.0001. (**c**–**k**) Representative electron microscopy images of lamellar bodies in hiPSC-derived AT2 cells (white arrow heads **c**,**d**) of lamellar bodies showing classical lamellar structures (**e**)**.** Typical microvilli structures at the surface of the cells (**f**) and characteristic multivesicular bodies indicated with white arrow heads (**g**). Tight cell–cell desmosome contacts indicated by the white arrow heads (**h**). (**i**–**k**) Apical tight junctions indicated by white arrow heads. M = mitochondria; N = nucleus; LB = lamellar body; MV = microvilli; MB = multivesicular body. Scale bar = 500 nm; magnification scale bars = 100 nm. (**l**–**o**) Exemplary surfactant expression in ALI versus submerged conditions (**l**,**m**) Immunofluorescence staining of SFTPB at day 27 of differentiation of hiPSC-derived AT2-like cells cultured under submerged or ALI conditions, respectively. (**n**,**o**) Immunofluorescence staining of SFTPC at day 27 of differentiation of hiPSC-derived AT2-like cells cultured under submerged or ALI conditions, respectively. Scale bars = 400 µm. (**p**) Comparison of submerged and ALI cultures, based on semi-quantitative analysis of SFTPB and SFTPC expressing cells.

Trans-Epithelial Electrical Resistance (TEER) measurements confirmed the formation of tight lung epithelial structures in hiPSC-derived AT2-like cells. As shown in Fig. 4a, the integrity of the formed lung epithelium was significantly established after 7 days of ALI culture in the 96-Transwell plates (1189; [9489, 1502] Ω × cm^2^, p < 0.003). In comparison to more proximal cell types like human small airway epithelial cell (hSAEC) cultures AT2-like cells showed significantly higher TEER function (p < 0.0001). Evaluation of AT2-like TEER values to literature data of human primary alveolar epithelial cells, showed no significant difference (p < 0.7345), (Fig. 4b). For statistical details see Supplementary Tables S16 and 17, online. Furthermore, the exposure of the AT2-like cells to the air through the ALI conditions supported the maturation of cells, as SFTPB and SFTPC expression exemplarily showed (Fig. 4l–o). As depicted in Fig. 4p semi-quantitative analysis showed a significant increase in both, SFTPC and SFTPB cell populations under ALI conditions (SFTPC: p = 0.0007; SFTPB: p = 0.001). Additionally, transmission electron microscopy (TEM) images clearly showed the presence of AT2 specific lamellar bodies, microvilli (MV), dense apical tight junctions, desmosomes and multi vesicular bodies (Fig. 4c–k). This indicates that functional AT2-like cells were efficiently generated during the maturation phase (see also Supplementary Fig. S4a–d). The hiPSC-derived AT2-like cell cultures were also analyzed regarding other endodermal phenotypes. No similarities were detected between AT2-like cells and hepatocytes (Alpha-1-Fetoprotein expression^51,52^), thyroid-like cells (PAX8 expression in LPCs) or proximal airway markers (see Supplementary Figs. S5f, S6, S13, online).

The here established generation of functional monolayer AT2-like cells out of hiPSCs was also reproducible in two alternative hiPSC lines (see Supplementary Figs. S7–10, online).

### Functional analysis of hiPSC-derived AT2-like cells in 96-Transwell format and subsequent changes in pro-fibrotic marker expression levels

**Figure 5:**
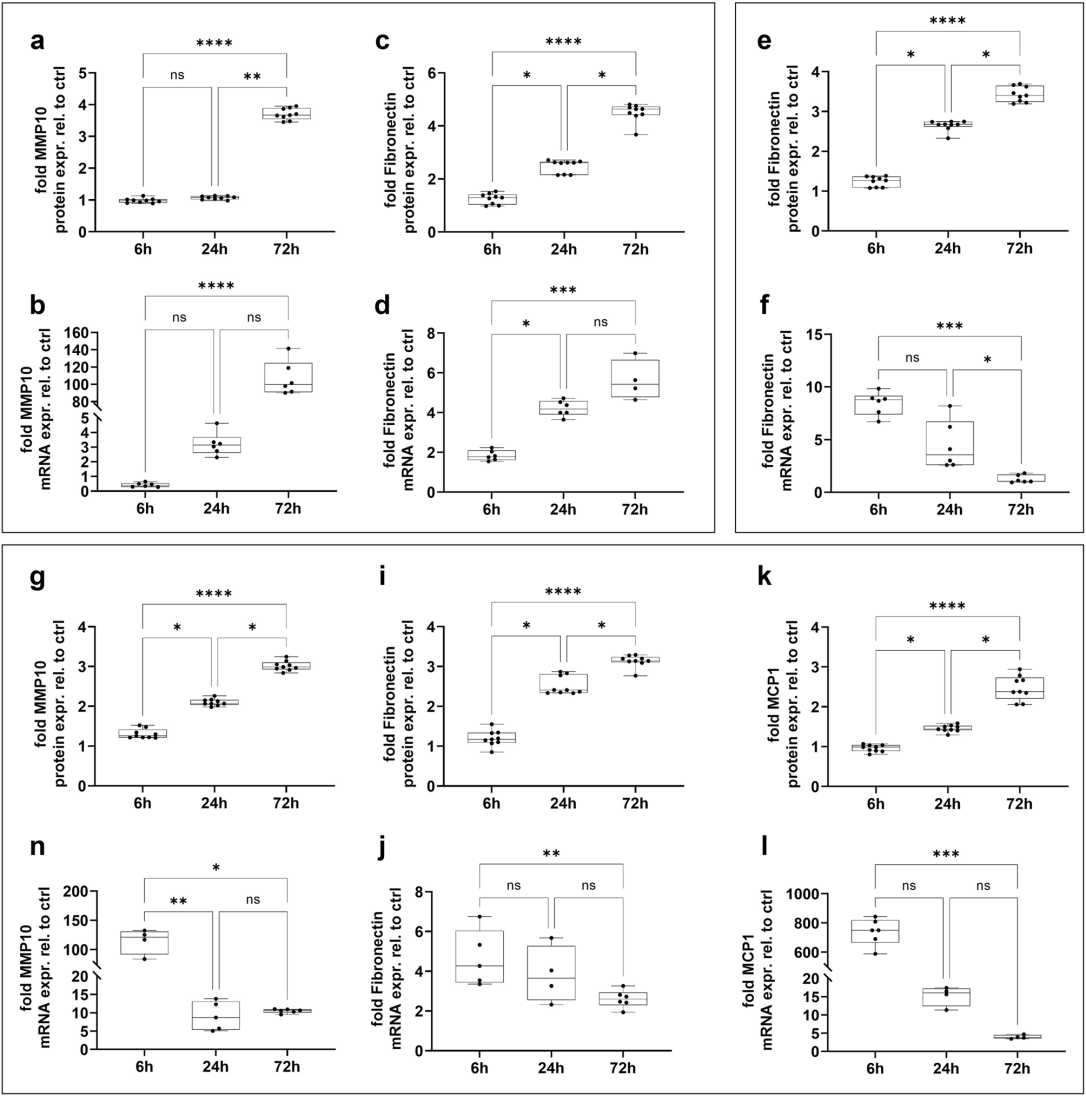
Physiological changes in transcriptional and secretory levels of known pro-fibrotic proteins caused by stimulation of hiPSC-derived alveolar like 2 cells in the presence of different cytokines and growth factors. (**a**–**d**) Time-dependent relative expression and secretion of MMP10 (**a**,**b**) and Fibronectin (**c**,**d**) on protein and mRNA level due to 10 ng/mL TGFβ-1 stimulation, normalized to unstimulated control cells. (**e**,**f**) Time-dependent relative expression of Fibronectin on protein and mRNA level due to 10 ng/mL IL-1β stimulation, normalized to unstimulated control cells. (**g**–**l**) Time-dependent relative expression of MMP10 (**g**,**h**), Fibronectin (**i**,**j**) and MCP-1 (**k**,**l**) on secreted protein and mRNA level due to 10 ng/mL TNF-α stimulation, normalized to unstimulated control cells. Median; range [min, max], N = 3; nd = not detectable, ns = not significant, *p < 0.05, **p < 0.01, ***p < 0.001, ****p < 0.0001.

Transforming growth factor β1 (TGF-β1), Tumor-Necrosis-Factor-α (TNF-α) and Interleukin-1β (IL-1β) are key mediators of IPF disease pathogenesis^53,54^. The respective cytokine challenges were replicated in vitro by administration of cytokines and growth factors to ALI-matured hiPSC-derived AT2-like cells in 96-Transwell plates (Fig. 5). In order to monitor the cellular response to the respective stimulus, changes in secretion and expression levels of distinct pro-fibrotic proteins were measured over the period of 72 hours (h).

In IPF, TGF-β1 is a key inducer of Metalloproteinase (MMP) expression besides other pro-fibrotic biomarkers. Specifically, MMP10 is known to correlate with disease severity^55,56^. Concurring with the human situation, a significant time-dependent increase in MMP10 expression, both on protein (p < 0.0001) and mRNA level (p < 0.0001) was observed (Fig. 5a,b). Additionally, TGF-β1 is known to induce elevated levels of fibronectin deposition^57^. In agreement with the in vivo pathogenesis, TGF-β1 was found to significantly increase the secretion of fibronectin (p < 0.0001), as well as the expression on mRNA level (p < 0.0005) from AT2-like cells (Fig. 5c,d).

Similar results were found in stimulation experiments with IL-1β. Protein expression levels of fibronectin were significantly increased over time (p < 0.001), whereas on gene expression level 8.8-fold higher expression levels were detected 6 h after stimulation compared to control cells (8.78; [6.72, 9.84]) (Fig. 5e,f).

TNF-α has also been shown to induce the secretion of MMP10, fibronectin, as well as the Monocyte chemoattractant protein-1 (MCP-1) in IPF disease pathology^58–60^. Reflecting this in vivo biology in the 96-Transwell plates, TNF-α induced ALI matured AT2-like cells to significantly increase the release of MMP10, fibronectin and MCP-1, with highest secretion after 72 h of stimulation (p < 0.0001) (Fig. 5g,i,k). Likewise, highest mRNA expression levels were detected 6 h after stimulation with 120-fold (*MMP10*), fourfold (*fibronectin*) and 700-fold (*MCP-1*) higher expression levels compared to control cells (Fig. 5h,j,l). Detailed statistics for all stimulation experiments are provided in Supplementary Tables S18–29, online. To exclude any cytotoxic effects, a LDH (Lactate dehydrogenase) release assay was performed over the time course of 72 h. No significant differences in comparison to vehicle controls were observed, supporting the suitability of the model to serve as a physiological relevant assay system to study epithelial dysfunction in lung diseases (see Supplementary Fig. S5j, online).

## Discussion

This study implemented a novel medium throughout, 2D monolayer-like, hiPSC-derived 96-Transwell ALI model of alveolar epithelial cells, to investigate epithelial dysfunction. This work demonstrates the suitability of the model to serve as a physiological assay system to study the effects of IPF-relevant cytokines and growth factors regarding the secretion of IPF associated biomarkers.

As working with hiPSCs is still an emerging field, other studies have also reported the generation of AT2-like cells, based on both embryonic and induced pluripotent stem cells^35,43,46,50,61–65^. However, protocols suitable for drug development and medium throughput applications have not been reported yet. The differentiation protocol established in this study followed these published strategies, but also included major modifications and significant improvements regarding cell banking and the decoupling of subsequent maturation of cells in 96-Transwell ALI cultures.

In fact, the objectives of this work were the following: (1) establishing an optimized differentiation protocol generating robust DE cells and the subsequent cryopreservation (2) generating LPCs and the following cell banking step as a major intermediate stage of differentiation (3) the decoupling of the final maturation process into alveolar-like cells in miniaturized 96-Transwell ALI cultures and (4) the application of the AT2-like cells to study epithelial dysfunction in IPF, as a canonical example to establish this highly physiological in vitro model.

First, an optimized stepwise differentiation of hiPSCs into DE cells with respect to DE cell amplification was described, based on a previously published protocol^40^. The produced DE cells were demonstrated to express classical DE markers on the gene expression and protein level. Furthermore, cryopreservation of DE cells could be established, maintaining their differentiation potential towards subsequent AFE stage, highlighted by clear DE marker expression after the cryopreservation and comparable SOX2^+^ and FOXA2^+^ AFE cells after 10 days of differentiation. The capability of the rethawed DE cells to differentiate into AFE cells corroborates the feasibility of the cryopreservation step.

Second, another major advantage regarding upscaling compatibility was the cryopreservation step on the level of lung progenitor cells. Based on cryopreserved DE cells, the generation of NKX2.1^+^/CPM^+^/SOX9^+^ LPCs was achieved within 21 days of differentiation. In line with other published protocols, a reproducible homogeneity of 73–89% in LPC cultures prior to cryopreservation was achieved (based on CPM and NKX2.1 staining)^35,50^. In contrast to several other protocols^35,46,50,66,67^, no sorting of cells prior to maturation was necessary. As the following analysis showed, this was an optimal condition to generate the desired AT2-like cells. Notably, based on the here presented work, the number of cells increased by a factor of 4.8-fold over the differentiation from hiPSCs to LPCs. This offers the possibility to use this highly physiological model for typical compound optimization campaigns in medicinal chemistry, where huge cell numbers are required.

The generation of stem cell-derived lung cells is one of the most complex endeavors due to the multistage embryology of the human lung. In comparison to the differentiation of other cell types, the within this study established 6-step differentiation protocol is complex and difficult to implement^68–70^. Therefore, the cryopreservation steps of DE cells and LPCs is an innovative, time-saving approach, so that such a highly physiological in vitro model can be provided in a reasonable format for upscaled applications in drug discovery research.

Third, key properties of medium throughput applications are the automation as well as the miniaturization of drug discovery assays. The decoupling of the final maturation of LPCs into AT2-like cells in 96-Transwell plates, serves these requirements. Classical Transwell cultures with human primary cells or stem cell- derived cells were mainly maintained in 24- or 12-Transwell plates and are therefore highly limited in their throughput. The here established 2D monolayer-like cultures provide easy morphological tracking of cells throughout the differentiation process and do not require complex 3D arrangements regarding spheroid and organoid formation. Despite these 3D applications are widely used even in the lung field, they have their limitations concerning variability of spheroid size, morphology and accessibility to cells in their matrices^71,72^. In contrast to the 3D arrangements, the ALI conditions enable the exposure of the AT2-like cells to the air, mimicking even more the human physiological situation. Additionally, downstream applications such as imaging and lysis of cells for PCR analysis is easier to implement and more convenient to work with in the 2D monolayer-like cell cultures. In addition, the miniaturization and the 2D monolayer-like ALI culture approach leads to a reduction in consumables and cost savings and simultaneously leads to a higher throughput. Furthermore, this 96-Transwell system is applicable to a robotic system^73^ to reduce the required manual working capacity and therefore provides a medium throughput platform towards pharmaceutical drug discovery.

While ALI cultures have been applied to primary human airway cells and hiPSC-derived airway epithelial cells to enhance maturation, this method had previously not been tested in a miniaturized 96-Transwell format^46,61,74–76^. To mimic the physiological situation of the alveolar epithelium in vitro, cells were exposed to the air through the ALI conditions. This supported the maturation of cells, as ratios of SFTPC and SFTPB in submerged versus ALI cultures showed. Pulmonary surfactants are crucial components of the alveolar epithelium, since they lower surface tension^77^ and consequently many studies rely on the expression on protein and mRNA level of *SFTPC* and *SFTPB* to determine the AT2 phenotype^35,50,64,78^. As highlighted in the results, *SFTPC* and the lamellar body associated protein *LPCAT-1* are significantly higher expressed compared to HPAEpiC, underlying the efficient generation of the disease relevant AT2-like cells in a physiological manner. This is in line with the clear presence of lamellar bodies in TEM images, AT2 specific surfactant producing and storing organelles^79,80^. Likewise, the presence of AT1 cells, was demonstrated in the here described alveolar-like cell cultures. No significant differences in marker expression levels of matured day 27 cell cultures compared to HPAEpiC were detected regarding AT1 markers, emphasizing the physiological composition and relevance of the model. Furthermore *TM4SF1*, a marker for alveolar epithelial progenitor cells within the adult human lung, is present in the AT2-like cultures^81^. Remarkably, the generated AT2-like cells stain positive for ABCA3, a marker which is not detectable in fetal lungs prior to 22–23 weeks of gestation, again underlying the physiology of the cells^82^. Additionally, the proper epithelial barrier function was confirmed by the expression of different tight junction proteins, as wells as the presence of strong cell–cell contacts in TEM images. Moreover, the generated AT2-like cells show a strong polarization with classical microvilli at the apical surface of the cells, which is in line with physiological TEER values after 1 week of ALI culture. In comparison to primary human alveolar cells, no significant difference in TEER values was detected, highlighting the physiological relevance of the cell model.

Due to the plastic differentiation potential of hiPSCs, the within this work generated hiPSC-derived AT2-like cell cultures were also analyzed regarding other endodermal phenotypes. No similarities were detected, underlying the efficient generation of mature AT2-like cells. Thus, despite the complex differentiation protocol and the difficulties of the maturation of cells in miniaturized 96-Transwell ALI cultures, these results confirm, that this model closely resembles the human physiological situation in vitro*.*

Fourth, the here established model of hiPSC-derived AT2-like cells was applied to study epithelial dysfunction in IPF. The pathogenesis of IPF is very complex and the disease-specific milieu within the lung has been suggested to play a central role in the development and progression for the disease^83,84^. TGF-β1 is known to be a key disease mediator^85–87^. In IPF lungs, TGF-β1 promotes the release of other pro-inflammatory molecules and induces the secretion of pro-fibrotic markers, such as extracellular matrix (ECM) components, e.g. collagen or fibronectin^7^. In agreement with that, the stimulation of AT2-like cells with 10 ng/mL of the TGF-β1 lead to a significant secretion of the pro-fibrotic marker protein fibronectin over the time course of 72 h. Apart from TGF-β1, also TNF-α and IL-1β play important roles in IPF pathogenesis^54,88,89^. Similarly, to TGF-β1, TNF-α and IL-1β both induce the release of the ECM protein fibronectin, verified by protein secretion and mRNA expression levels. These cytokines are also known to induce an overproduction of MMPs, such as MMP10^90^, as well as pro-inflammatory chemokines such as MCP-1^89,91,92^. Correspondingly, TNF-α and TGF-β1 induced the release of MMP10, whereas TNF-α additionally stimulated the release of MCP-1. Taken together, the iPSC-derived in vitro model reacts in a highly physiological manner and therefore resembles certain IPF-related changes in epithelial transcription and secretion levels of pro-fibrotic marker proteins.

Of note, in cells stimulated by TNF-α or IL-1β, the first 6 h showed the highest mRNA levels of pro-fibrotic markers. This contrasts with TGF-β1 stimulated cells, where marker mRNA levels were still significantly increasing over the time of 72 h. The underlying differences could be based on the different signaling pathways, mRNA turnover and subsequent feedback loops trigged by different cytokines and growth factors. TGF-β1 mainly signals through Smad signaling in fibrotic diseases^93,94^, whereas TNF-α had earlier been recognized to stimulate nuclear factor kappa B (NF-κB)-mediated signaling^4,95^. IL-1β is known to trigger intracellular signaling cascades that induce as well the NF-κB signaling, leading to the expression of pro-inflammatory cytokines, chemokines, and secondary mediators of the inflammatory response^96,97^. Following, the turnover of mRNA transcripts and subsequent protein translation in these signaling pathways may differ. This corresponds well with the observation in mRNA expression levels in IL-1β and TNF-α stimulated AT2-like cells compared to their corresponding protein expressions.

As a complex cellular interplay leads to the pathogenesis of IPF^98^, the here developed model system enables a perfect platform for co-cultures, such as fibroblasts, immune cells and endothelial cells to better understand the role of a pro-fibrotic extracellular milieu in such a complex physiologically relevant setting. The implemented 96-Transwell model also opens new routes to novel therapeutic concepts related to recovery studies of epithelial barrier function. The innovative TEER device avoids inconsistent data acquisition and serves as quality control of cells after differentiation. It also could be used in the established set-up for medium throughput studies regarding dose–response curves of stimuli and possible recovery studies of epithelial barrier function. This has already been successful in different lung cell cultures but has never been transferred to hiPSC-derived cell models^95,99–101^.

Regarding future work, some topics should be addressed in more detail. Cryopreservation steps were implemented at day 6 and day 21 of differentiation. These two specific time points were selected, based on proliferation potential of cells and the determination of the germ layer (DE cells) and subsequent specification towards lung pattern (LPCs) already took place. To realize even higher cell numbers in frozen stocks to serve in drug discovery campaigns, further experiments concerning the expansion potential of DE cells should be performed. An optimization step would be to positively influence the viability of DE cells after cryopreservation through a spectrum of supplements^102–104^.The resulting even higher DE cell numbers and subsequent aliquoted frozen cell stocks could be beneficial to serve as a basis for regularly repeated drug discovery assay cycles. Additionally, the establishment of an expansion step for the LPCs through the administration of growth factors, as it was already implemented in neuronal progenitor cell differentiation protocols^105^, could further increase the yield of cells.

In addition, the here generated progenitor cells could be also used as a starting point for other complex differentiation processes, such as hepatocyte-like cells or thyroid cells, both in drug discovery and basic research. Another major advantage of using hiPSCs as a basis for the development of such a highly physiological in vitro model is, that it offers the possibility to generate different cell types out of the same cellular and genetic origin, enabling possible co-cultures and future personalized medicine approaches^106^. While there is substantial evidence for the relevance of various cytokines and growth factors in IPF, including TGF-β1, TNF-α and IL-1β, no single factor is known to simultaneously activate all IPF relevant pathways^83,89^. To better mimic the complexity of the pro-fibrotic milieu in vitro, this model can be used for further experiments and evaluations regarding the effects of a cytokine cocktail on the hiPSC-derived AT2-like cells^45^.

In summary, this work has established an hiPSC-derived and miniaturized AT2-like cell model system, which provides a unique opportunity to study IPF in vitro. The model is based on a monolayer-like differentiation protocol (without intermediate 3D steps). The combination of cryopreservation steps, the decoupling of the maturation in 96-Transwell cultures and a possible robotic integration enables a medium throughput approach towards pharmaceutical drug discovery. This technical innovation described in this study, opens new routes for target-focused and phenotypic drug discovery as well as basic research.

## Methods

### Differentiation of hiPSCs towards alveolar epithelial type 2 like cells

#### Human induced pluripotent stem cells maintenance

Either Cellartis Human hiPSC Cell Line 18 (ChiPSC18; cat. Y00305; Takara Bio Europe AB; Goteborg, Sweden) or Cellartis Human hiPSC Cell Line 22 (ChiPSC22; cat. Y00325; Takara Bio Europe AB; Goteborg, Sweden) was cultured and maintained according to manufacturer’s instructions in feeder free conditions. Briefly, cells were cultured on COAT-1 (cat. Y30012, Takara Bio Europe AB; Goteborg, Sweden) coated cell culture flasks in a humidified atmosphere of 5% CO_2_ at 37 °C with a daily media change using freshly prepared Cellartis DEF-CS 500 complete medium (Cellartis DEF-CS 500 Basal Medium (cat. Y30011; Takara Bio Europe AB), supplemented with DEF-CS GF1 (diluted 1:333; cat. Y30016; Takara Bio Europe AB) and DEF-CS GF2 (diluted 1:1000; cat. Y30016; Takara Bio Europe AB)). DEF-CS GF3 (diluted 1:1000; cat. Y30016; Takara Bio Europe AB) was only added to Cellartis DEF-CS 500 complete medium when hiPSCs were thawed, passaged or cryopreserved and was not used for regular medium changes.

Prior to cell seeding, cell culture flasks were coated with 0.1 mL/cm^2^ of COAT-1 diluted 1:20 in 1× DPBS (+/+) (cat. 14040083, Thermo Fisher Scientific, Waltham, MA) for at least 30 min (min) at 37 °C, 5% CO_2_. Cryopreserved hiPSCs were seeded at 1.6 × 10^5^ cells per cm^2^ in Cellartis DEF-CS 500 complete medium and were cultured for at least 3 days until first passaging after thawing.

After reaching 80% confluence, cells were washed once with 1× DPBS (−/−) (cat. 14190144, Thermo Fisher Scientific, Waltham, MA) and detached using 40 µL/cm^2^ of 1× TrypLE Select Enzyme (cat. 12563-011; Life Technologies; Carlsbad, CA). After an incubation time of up to 7 min at 37 °C, the flask was rinsed with 10 volumes of Cellartis DEF-CS 500 complete medium. Cells were replated at a density of 4 × 10^4^ cells per cm^2^ (4 days culture) or at 5 × 10^4^ cells per cm^2^ (3 days culture) in Cellartis DEF-CS 500 complete medium. For cryopreservation, the flask was rinsed with 5 volumes of Cellartis DEF-CS 500 complete medium after detaching. Cells were centrifuged at 200*g* for 5 min and resuspended at 6.5 × 10^6^ cells per mL in STEM-CELLBANKER (cat. 11890; Amsbio; Abingdon, UK) for long-term storage.

#### Differentiation of hiPSC into alveolar epithelial type 2 like cells

Prior to the start of differentiation, the hiPSC were expanded and the DE formation was performed as follows^40^: Cell culture flasks were coated with Matrigel (cat. 354277; Corning, Corning, NY) diluted in 1× DPBS according to the manufacturer’s instructions for one hour at 37 °C. Subsequently, hiPSCs were detached as described for maintenance, using TrypLE Selected Enzyme. The single cell suspension was seeded at a density of 0.1 × 10^6^ cells/cm^2^ in DE medium day 0 (composition see Table 1). The medium was changed daily until day 6 using the specific composition according to Table 1.

**Table 1:** DE medium composition during the phase of DE differentiation.

Composition	Cat #, supplier	Stock [c]	Final [c]	
RPMI1640	11875-093, Thermo Fisher	1	1	
B27 supplement	17504-044, Thermo Fisher	50×	1×	
Penicillin–streptomycin	15140-122, Thermo Fisher	10,000 U/mL	50 U/mL	
Activin A	338-AC-050, Tocris	100 µg/mL	0.1 µg/mL	*d0-6*
CHIR99021	CT 99021, Axon Medchem	3000 µM	1 µM	*d0-6*
Y-27632	Ab120-129, Abcam	10,000 µM	10 µM	*d0-1*
Sodium butyrate	303410-100G, Sigma	25.00 mM	0.25 mM	*d1*
Sodium butyrate	303410-100G, Sigma	25.00 mM	0.125 mM	*d2-6*

After 6 days, obtained DE cells were cryopreserved. Therefore, the cells were harvested by washing the cells once with 1× DPBS and by adding 1× TrypLE Select Enzyme. After an incubation time of 5 min at 37 °C, the flask was rinsed with RPMI1640 basal medium (cat. 11875-093; Thermo Fisher Scientific) and cells were centrifuged at 200*g* for 5 min. The pellet was resuspended to a final concentration of up to 6.5 × 10^6^ cells per mL in cooled CryoStor CS10 (cat. 7959; Stemcell Technologies; Vancouver, Canada), supplemented with 10 µM Y-27632 dihydrochloride (ROCK inhibitor; cat. ab120129; Abcam; Cambridge, UK) for long-term storage.

Thawing of cryopreserved DE cells for subsequent differentiation into Anterior Foregut Endoderm (AFE) was performed in RPMI1640 basal medium. Cell suspension was collected, centrifuged at 200*g* for 5 min, resuspended in DE medium day 6, supplemented with 10 µM ROCK inhibitor, and plated on freshly coated Matrigel plates. Importantly, DE cells were seeded in the same density compared to the DE cells harvested prior to cryopreservation in Matrigel-coated cell culture ware.

The next day, the DE cells were differentiated into AFE cells by replacing the DE medium with AFE medium for 4 days (day 6–day 10, see Table 2). Differentiation was forged ahead towards ventralized AFE (vAFE) cells using the vAFE medium (see Table 3) from day 10 to 14. In order to push the vAFE cells into lung epithelial progenitor cells, the progenitor medium (see Table 4) was used from day 14 to day 21.

**Table 2:** AFE medium composition during the phase of AFE differentiation*.*

Composition	Cat #, supplier	Stock [c]	Final [c]	
DMEM/F12, GlutaMAX	10565018, Thermo Fisher	1	1	
B27 supplement	17504-044, Thermo Fisher	50×	1×	
N2 supplement	17502-048, Thermo Fisher	100×	1×	
Penicillin–streptomycin	15140-122, Thermo Fisher	10,000 U/mL	50 U/mL	
l-Ascorbic acid	A4403-100MG, Sigma	5 mg/mL	0.05 mg/mL	
Mono-thioglycerol (MTG)	M6145, Sigma; pre-dilute 1:10 in DMEM/F12	1150 mM	0.4 mM	
Noggin	3344-NG-050, Tocris	100.00 µg/mL	0.1 µg/mL	*d6-10*
SB431542	1614, Tocris	10.00 mM	0.01 mM	*d6-10*

**Table 3:** vAFE medium composition during the phase of vAFE differentiation.

Composition	Cat #, supplier	Stock [c]	Final [c]	
DMEM/F12, GlutaMAX	10565018, Thermo Fisher	1	1	
B27 supplement	17504-044, Thermo Fisher	50×	1×	
N2 supplement	17502-048, Thermo Fisher	100×	1×	
Penicillin–streptomycin	15140-122, Thermo Fisher	10,000 U/mL	50 U/mL	
l-Ascorbic acid	A4403-100MG, Sigma	5 mg/mL	0.05 mg/mL	
Mono-thioglycerol (MTG)	M6145, Sigma; pre-dilute 1:10 in DMEM/F12	1150 mM	0.4 mM	*d10-14*
BMP4	314-BP, Tocris	10,000 ng/mL	20 ng/mL	*d10-14*
CHIR99021	CT 99021, Axon Medchem	3000 µM	3,5 µM	*d10-14*
ATRA	R2625-1G, Sigma	1000 µM	1.0 µM	*d10-14*

**Table 4:** Lung progenitor medium composition during the phase of lung progenitor differentiation.

Composition	Cat #, supplier	Stock [c]	Final [c]	
DMEM/F12, GlutaMAX	10565018, Thermo Fisher	1	1	
B27 supplement	17504-044, Thermo Fisher	50×	1×	
N2 supplement	17502-048, Thermo Fisher	100×	1×	
Penicillin–streptomycin	15140-122, Thermo Fisher	10,000 U/mL	50 U/mL	
l-Ascorbic acid	A4403-100MG, Sigma	5 mg/mL	0.05 mg/mL	
Mono-thioglycerol (MTG)	M6145, Sigma; pre-dilute 1:10 in DMEM/F12	1150 mM	0.4 mM	*d14-21*
FGF7 (KGF)	251-KG, Tocris	10 µg/mL	0.01 µg/mL	*d14-21*
DAPT	72082, STEMCELL Technologies	1000 mM	0.02 mM	*d14-21*
FGF10	345-FG, Tocris; 1/10 in DMEM/F12 pre-diluted	10 µg/ml	0.01 µg/ml	*d14-21*
CHIR99021	CT 99021, Axon Medchem	3000 µM	3.0 µM	*d14-21*

After 21 days of differentiation, obtained lung progenitors were cryopreserved. Therefore, the cells were harvested by washing the cells once with 1× DPBS and by adding 80 µL/cm^2^ of 1× TrypLE Select Enzyme. After an incubation time of 10 min at 37 °C, the wells were rinsed with DMEM/F12 basal medium (cat. 10565018; Thermo Fisher Scientific) and cells were centrifuged at 200*g* for 5 min. The pellet was resuspended in cooled CryoStor CS10, supplemented with 10 µM ROCK inhibitor for long-term storage.

Importantly, for optimal lung progenitor thawing, the cell number per vial was determined by the following equation:

$$X= \frac{\left(\frac{total \; live \; cell \; number}{Z}\right)*Y}{2}$$

with X [cells] = cells per vial for cryopreservation for thawing in Y; Y [cm^2^] = cell culture format for lung progenitor thawing and Z [cm^2^] = cell culture format of differentiation.

To generate alveolar epithelial cells, the cryopreserved lung progenitors were further cultured in 96-Transwell plates (cat. 3342; Corning; Corning, NY) in a submerged manner at day 21. Prior to cell seeding, filters of 96-Transwell plates were pre-coated with 50 µL of Matrigel for one hour at 37 °C and 5% CO_2_. Remaining coating solution was aspirated after the incubation time. Thawing of cryopreserved cells for subsequent alveolarization of lung progenitor cells was performed in lung progenitor medium (see Table 4). Cell suspension was collected, centrifuged at 200*g* for 5 min and resuspended in progenitor medium, supplemented with 10 µM ROCK inhibitor. Then, 1 × 10^5^ cells/well in 50 µL/well were dispensed into the Matrigel-coated apical chambers of the 96-Transwell plates, containing 180 µL/well of alveolarization medium, supplemented with 10 µM ROCK inhibitor in the basal compartment. 24 h (h) after thawing (day 22), medium was replaced with freshly prepared progenitor medium (w/o ROCK inhibitor). At day 23, medium was changed to alveolarization medium (see Table 5), supplemented with 10 μM ROCK inhibitor, followed by a medium change on day 24 without ROCK inhibitor. The air–liquid interface condition was established at day 24 (3 days post-seeding) by the complete aspiration of the medium in the apical compartment, and cells were cultured until day 27 (stimulation of cells) or day 30.

**Table 5:** Alveolarization medium composition during the phase of final alveolar differentiation.

Composition	Cat #, supplier	Stock [c]	Final [c]	
Ham’s F12	21765029, Thermo Fisher	1	1	
Penicillin–streptomycin	15140-122, Thermo Fisher	10,000 U/mL	50 U/mL	
HEPES	15630080, Thermo Fisher	1000 mM	15 mM	
BSA	Sigma	2.50%	0.25%	
Ca2Cl	Sigma	1000.0 mM	0.8 mM	
ITS premix	354350, Corning	100.00%	0.10%	
B27	17504-044, Thermo Fisher	50×	1×	
IBMX	I5879-100MG, Sigma	100.0 mM	0.1 mM	*d21-30*
8-Br-cAMP	B5386-25MG, Sigma	50.0 mM	0.1 mM	*d21-30*
Dexamethasone	D4902-100MG, Sigma	50.00 µM	0.05 µM	*d21-30*
FGF7 (KGF)	251-KG, Tocris	10 µg/mL	0.01 µg/mL	*d21-30*
Y-27632	Ab120-129, Abcam	10,000 µM	10 µM	*d21-30*

### Measurement of epithelial barrier integrity

Trans-epithelial electrical resistance (TEER) over the epithelial layer in the 96-Transwell plates was measured at the end of the differentiation process (day 27) in order to analyze the integrity of the epithelium. Measurement was conducted as described in detail in Bluhmki et al.^73^, using an automated 96-electrode device. Shortly, prior to the TEER measurements, cells were washed once with pre-warmed 1× DPBS to remove possibly produced mucus. To allow for the electrical measurement, 120 µL/well of pre-warmed medium was added subsequently to the apical chambers of the Transwell plate. Correction of raw data was accomplished by subtracting the electrical resistance as measured over an empty, cell-free insert. The final TEER values were obtained by multiplying with the insert area of the synthetic Transwell filter.

### Immunofluorescence staining of markers for different cell stages during differentiation

Marker proteins of different cell stages during the differentiation process from hiPSCs towards AT2-like cells were visualized by immunolabeling and confocal microscopy. For this purpose, the cells were washed once with 1× DPBS and subsequently fixed with 4% (v/v) paraformaldehyde solution (cat. 252549-500 ml; Sigma-Aldrich; St. Louis, MO) for 15 min at room temperature. Cells cultured on filters of 96-Transwell plates were fixed by adding 50 µL/well of 4% PFA in the apical compartment and 180 µL/well in the basal compartment. After three washing steps with 1× DPBS, the cells were permeabilized with 0.3% (v/v) Triton X-100 (cat. T8787-100 ml; Sigma- Aldrich) in 5% (w/v) Bovine Serum Albumin (BSA) (cat. A3059-100G; Sigma-Aldrich) in 1× DPBS for 60 min at room temperature. This permeabilization step was skipped for the cell membrane proteins EPCAM, CD184 (CXCR4) and CPM. Subsequently, the cells were washed three times with 1× DPBS, then incubated at 4 °C over night with the indicated primary antibodies (see Table 6 of antibodies and respective dilution factors) and Hoechst 33342 (cat. H3570; Thermo Fisher Scientific; diluted 1:5000) diluted in 1% (v/v) BSA in 1× DPBS. The next day, the cells were washed three times with 1× DPBS and then incubated in the dark for 2 h at room temperature with species-specific secondary Alexa Fluor antibodies (see Table 7), supplemented with 1% (v/v) BSA in 1× DPBS. Finally, the cells were washed three times with 1× DPBS. Membranes of Transwell plates were removed from the plastic support for ensuing mounting on a microscopic slide using the ProLong Diamond Antifade Mountant (cat. P36961, Thermo Fisher Scientific). Imaging of immunolabeled cells was performed using a LSM710 laser confocal microscope (Carl Zeiss Microscopy, Jena, Germany).

**Table 6:** List of primary antibodies used in this study.

Antibody	Cat. no.	Vendor	Dilution
ABCA3	WRAB-70565	Seven Hills	1:1000
CAV1	ab2910	Abcam	1:500
CD184 (CXCR4)	60089	Stemcell Technologies	1:50
CPM	014-27501	WAKO	1:200
E-Cadherin	13-1700	Invitrogen	1:200
EPCAM	ab20160	Abcam	1:250
FOXA2	Af2400	R&D Systems	1:50
Hoechst33342	H3570	Thermo Fisher	1:5000
NKX2.1	WRAB1231	Seven Hills	1:1000
PAX8	ab53490	abcam	1:200
SFTPB	WRAB-48604	Seven Hills	1:1000
SFTPC	WRAB-9337	Seven Hills	1:2000
SOX17	AF1924	R&D Systems	1:250
SOX2	#9656	Cell Signalling	1:50

**Table 7:** List of secondary antibodies used in this study.

Antibody	Cat. no.	Vendor	Dilution
Alexa 488 goat anti-mouse IgG1	A21121	Thermo Fisher	1:500
Alexa 488 goat anti-mouse IgG2a	A-21131	Thermo Fisher	1:500
Alexa 488 donkey anti-rabbit IgG	A21206	Thermo Fisher	1:500
Alexa 488 goat anti-rabbit IgG	A-11034	Thermo Fisher	1:500
Alexa 546 goat anti-rabbit IgG	A11010	Thermo Fisher	1:500
Alexa 546 rabbit anti-goat IgG	A-21085	Thermo Fisher	1:500
Alexa 546 donkey anti-goat IgG	A-11056	Thermo Fisher	1:1000
Alexa 647 goat anti-mouse IgG	A-21236	Thermo Fisher	1:250
Alexa 647 goat anti-mouse IgG2b	A21242	Thermo Fisher	1:500

### Semi-quantitative measurement of SFTPC^+^/ SFTPB^+^ areas in hiPSC-derived cultures

Immunofluorescence of fixed whole 96-Transwell inserts was performed as described above. Six randomly selected areas per insert were captured using a 20× Plan-Apochromat objective on a LSM 710 confocal microscope system (Zeiss, Oberkochen, Germany) with the following settings: Alexa Fluor 488 channel, 5% laser power, master gain 600, acquisition speed 9. To account for unevenness in specimen surface and thickness, Z-stack imaging of 25 vertical stacks per area was applied and maximum intensity projection was performed using the ZEN 2012 Black Edition software (Zeiss, Oberkochen, Germany). Image analysis was performed using the Fiji for ImageJ software (11). Each image was first converted to 8-bit format and then converted to binary at a threshold of 20–255. To avoid overlapping of structures, the watershed function was performed to accurately cut structures apart. Afterwards, nuclei/ surfactant positive areas were analyzed by using the 3D Objects counter tool in ImageJ. For Nuclei counting areas within 60 px were analyzed and summarized per image. Surfactant positive cells were counted based on > 100 px. For each analyzed Transwell insert, SFTPC positive cell counts were normalized to total cell number (based on nuclei counting) and the percentage of SFTPC positive cells in hiPSC-derived AT2-like cells at day 27 were calculated from the six selected areas.

### Flow cytometry

Flow cytometry analysis of CPM and PAX8 in lung progenitor cells was performed on day 21 of differentiation. Cryopreserved lung progenitor cells were thawed on Matrigel-coated well plates according to the described thawing procedure in advance and stained for CPM, NKX2.1 and PAX8 expression the following day. Therefore, cells were detached from the culture by adding 80 µL/cm^2^ prewarmed 1× TrypLE Select Enzyme. After an incubation time of 10 min at 37 °C, the wells were rinsed with DMEM/F12 basal medium (cat. 10565018; Thermo Fisher Scientific) and centrifuged at 200*g* for 5 min. Cell pellet was resuspended in 1× DPBS and transferred to FACS tubes. All subsequent washing steps were performed by applying ice cold wash buffer consisting of 0.1% BSA in 1× DPBS. Cells were washed and tubes stained for PAX8 were additionally permeabilized in 80% MeOH for 5 min, followed by 0.1% Tween-20 (Sigma-Aldrich, St. Louis, MO, US) in wash buffer for 10 min. Blocking was performed in 10% goat serum (Sigma-Aldrich, St. Louis, MO, US) in PBS for 10 min. Cells were washed and incubated in the respective antibody solution for 15 min on ice in the dark. Unstained controls were incubated in wash buffer, isotype control for CPM were incubated in Goat anti-mouse IgG2b Cross Adsorbed Secondary Antibody, Alexa Fluor647, (Ca. No. A21242, Thermo Fisher, 1:500 diluted in 100 µL/5 × 10^5^ cells 1× DPBS, 1% BSA). Tubes stained for isotype matching PAX8 and NKX2.1 were incubated in 1 µg/mL Alexa Fluor 488 conjugated rabbit IgG Isotype Control (Abcam, Cambridge, UK). Samples for CPM staining were incubated with primary antibody (anti-CPM, Cat.No. 014-27501, Wako 1:200 diluted in 100 µL/5 × 10^5^ cells 1× DPBS, 1% BSA). Samples for PAX8 staining were incubated with primary antibody (anti-PAX8, Cat.No. ab53490, abcam 1:200 diluted in 100 µL/5 × 10^5^ cells 1× DPBS, 1% BSA). Samples for NKX2.1 staining were incubated with primary antibody (anti-NKX2.1, Cat.No. WRAB1231, Seven Hills 1:500 diluted in 100 µL/5 × 10^5^ cells 1× DPBS, 1% BSA). Next, cell pellet was washed twice with 5–10 volumes of cell staining buffer (1× DPBS, 1% BSA) and centrifuged at 200*g* for 3 min. Then, the secondary antibodies for CPM (Goat anti-mouse IgG2b Cross Adsorbed Secondary Antibody, Alexa Fluor488, Ca. No. A21242, Thermo Fisher, 1:500 diluted in 100 µL/ 5 × 10^5^ cells 1× DPBS, 1% BSA), PAX8 and NKX2.1 (Goat anti-rabbit IgG Cross Adsorbed Secondary Antibody, Alexa Fluor488, Ca. No. A-11034, Thermo Fisher, 1:500 diluted in 100 µL/ 5 × 10^5^ cells 1× DPBS, 1% BSA) were added and incubated for 15 min on ice, light protected. Again, cells were washed twice with 5–10 volumes cell staining buffer (1× DPBS, 1% BSA). Cells were passed through a 70 µm cell strainer cap to prevent blocking of FACS lines by cell aggregates. Samples were measured on BD FACS Calibur flow cytometer (BD Biosciences, Franklin Lakes, NJ, US) and analyzed using the FACSDiva (BD Biosciences, Franklin Lakes, NJ, US) and the FlowJo (FlowJo LLC) software.

### Transmission electron microscopy

The transmission electron microscopy images of hiPSC-derived AT2-like cells were generated following standard procedures. Shortly, 96-Transwell inserts were pre-fixed for 1 h with 2.5% glutaraldehyde (EM grade) and 2% Paraformaldehyde 0.1% cacodylate buffer solution. The PET membrane with pre-fixed cells was then carefully removed from the Transwell tray and washed with a 0.1% cacodylate buffer solution for 30 min. Subsequently, the samples were transferred into an EM-TP Tissue processor (Leica Biosystems, Nussloch, Germany) for automated post-fixation, staining, dehydration and embedding. The samples underwent the following preparation steps: 20 min in 0.1% cacodylate buffer, 3 h in 2% Daltons osmiumtetroxide aq., 3 times 15 min in 0.1% cacodylate buffer solution, 15 min in 30% isopropanol, 30 min each in 30%, 50%, 70%, 90% and 100% isopropanol, and three times 1 h in 100% isopropanol. Following dehydration, sample infiltration with Epoxy resin was achieved as follows: 30 min in 50% isopropanol/ 50% EPON, 30 min in 33% isopropanol/ 66% EPON, 30 min in 20% isopropanol/ 80% EPON and 60 min in 100% EPON. Afterwards, the samples were then incubated twice for 6 h in 100% EPON and hardened at 60 °C for 24 h. Ultra-thin sections (50 nm) were prepared on an Ultracut UCT ultra-microtome (Leica Biosystems, Nussloch, Germany) and imaged on a TEM 912AB (Zeiss, Oberkochen,Germany).

### Gene expression profiles

For gene expression analysis, the Cells-to-CT 1-Step TaqMan Kit (cat. A25602; Invitrogen; Carlsbad, CA) was used according to the manufacturer’s instructions. In short, cells were washed with 25 µL of cold 1× DPBS and lysed with 25 µL DNase/Lysis Solution (dilution 1:100). After 5 min, 2.5 µL of stop solution were added and incubated for 2 min. Generated lysates were either stored at − 20 °C or were directly used in subsequent RT-PCR experiments. Total human pulmonary alveolar epithelial cell (HPAEpiC) RNA lysate was purchased from BioTrend (cat. 3205-SC) and processed the same way as cell culture lysates.

For the RT-PCR, 1 µL of lysate was added to a final reaction volume of 10 µL containing 2.5 µL of TaqMan 1-Step qRT-PCR Mix, 0.5 µL of the respective TaqMan Gene Expression Assay, 20× (cat. 4351370; FAM Dye; Thermo Fisher Scientific), as listed in Table 8, and 6 µL of Nuclease-free water (cat. AM9922; Invitrogen). Gene expression levels of individual genes were normalized to the reference gene encoding the RNA Polymerase II Subunit A (*POLR2A*). Calculated 2^(−Δct)^ values (expression relative to POLR2A) were plotted in analysis graphs. Fold expression levels were normalized to the reference samples (hiPSC or unstimulated cells) through the difference quotient of both 2^(−Δct)^ values.

**Table 8:** List of TaqMan Gene Expression Assays used within this study.

Target	Assay ID	Vendor
ABCA3	Hs00184543_m1	Applied Biosystems
CAV1	Hs00971716_m1	Applied Biosystems
CCL2 (MCP-1)	Hs00234140_m1	Applied Biosystems
CDH1	Hs01023895_m1	Applied Biosystems
cKIT	Hs00174029_m1	Applied Biosystems
CXCR4	Hs00237052_m1	Applied Biosystems
EPCAM	Hs00901885_m1	Applied Biosystems
FN1 (fibronectin)	Hs01549976_m1	Applied Biosystems
FOXA2	Hs00232764_m1	Applied Biosystems
ID2	Hs04187239_m1	Applied Biosystems
LPCAT1	Hs00227357_m1	Applied Biosystems
MIXL	Hs04400364_m1	Applied Biosystems
MMP10	Hs00233987_m1	Applied Biosystems
NKX2.1	Hs00968940_m1	Applied Biosystems
PDPN	Hs00366766_m1	Applied Biosystems
POLR2A	Hs00172187_m1	Applied Biosystems
POU5F1	Hs00999632_g1	Applied Biosystems
SFTPB	Hs00167036_m1	Applied Biosystems
SFTPC	Hs00161628_m1	Applied Biosystems
SOX17	Hs00751752_s1	Applied Biosystems
SOX2	Hs01053049_s1	Applied Biosystems
SOX9	Hs00165814_m1	Applied Biosystems
TM4SF1	Hs01547334_m1	Applied Biosystems

### Stimulation of cells

To investigate the physiological epithelial disruption of the generated alveolar epithelial type II like cells by a certain stimulus, the cells were stimulated with the indicated concentration of either TNF-α (cat. 210-TA-020; R&D Systems; Minneapolis, MN), IL-1β (cat. 201-LB-005/CF; R&D Systems) or TGFβ-1 (cat. 240-B; R&D Systems) at day 27 of LPC maturation. For that, both stimulants and negative vehicle controls were diluted in alveolarization medium and added to empty receiver plates (cat. 3382; Corning). Subsequently, 96-Transwell inserts from the cell plates were relocated to these receiver plates. Cellular stimulation was performed for 72 h at 37 °C and 5% CO_2_, with vehicle-treated cells serving as negative controls. After stimulation, the basal cell supernatants were collected and cells were washed on the apical side with 100 μL/well 1× DPBS to enable harvesting of apical supernatants. Cellular supernatants were frozen at − 20 °C until further analysis via time resolved fluorescence energy transfer (TR-FRET) for the expression of pro-fibrotic marker proteins, as described below.

### TR-FRET measurement of pro-fibrotic marker proteins

TR-FRET was used to measure the pro-fibrotic markers Fibronectin (LANCE assay), MCP-1 and MMP-10 (HTRF assays) in cell culture supernatants after stimulation of the epithelial cells as described above. The assay was performed according to the manufacturer’s instructions (CisBio and Perkin Elmer) in 384-well microplates (cat. 781075, Greiner Bio-One, Frickenhausen, Germany) with a total assay volume of 15 µL/well. In short, for the HTRF assays, sandwich pairs of Eu-cryptate- and d2-conjugated anti-target antibodies (cat. 62HCCL2PEH and 62MMP9PEH; Cisbio; Codolet, France) were diluted with PPI Buffer (cat. 61DB9RDF; Cisbio) as indicated in the respective instruction guide. 5 µL/well of these antibody solutions were combined with 10 µL/well of the supernatants, which were fivefold diluted in alveolarization medium for the MCP-1 measurement. For the LANCE assay, 2 µL/well of 1:5 diluted samples were transferred in an assay plate. Subsequently, 8 µL/well of Ultra HiBlock Buffer (cat. TRF1011C; PerkinElmer; Waltham, MA) and 2.5 µL/well of Eu-labeled anti-Fibronectin antibody (cat. TRF1351C; PerkinElmer) were added. After an incubation time of 30 min, 2.5 µL/well of U-Light-labeled anti-Fibronectin antibody (cat. TRF1351C; PerkinElmer) were added.

Some wells of the microplates received Fibronectin (cat. 4305-FNB; R&D Systems), MCP-1 (cat. 300-04; PeproTech, Rocky Hill, NJ) or MMP-9 (cat. 910-MP-010; R&D Systems) standard solutions instead of supernatants for the determination of absolute concentrations of these three pro-fibrotic marker proteins. These recombinant proteins were used to guarantee that the actual measurements were in the dynamic area of the standard curve. Plates were incubated for 2 h (fibronectin) or for 24 h (MCP-1, MMP-10) at room temperature and were measured using an EnVision-Reader (excitation: 320 nm; emission: 615 nm and 665 nm) from PerkinElmer. HTRF and LANCE ratio values were calculated as follows: ratio = 10.000 × emission @665 nm/emission @615 nm.

### Data analysis plan

Expression levels of different marker genes throughout the differentiation process presented in this work are depicted as mean with error bars representing the 95%CI (confidence interval) of at least three independent experiments. TEER measurements, one-step RT-PCR data of AT2-like cells and physiological stimulation data are shown as box blots with median and bars representing the minimum to maximum whiskers (median; [min, max]) of at least three independent experiments. Literature based TEER value of primary human alveolar lung cells was calculated based on published data listed in Supplementary Table S1, online. Statistical comparisons between groups were assessed by one-way ANOVA, followed by the Uncorrected Dunn’s test. Furthermore, TM4SF1 one-step RT-PCR data were statistically compared to a hypothetical value of 0 using the one-sample Wilcoxon signed rank test. Semi-quantitative analysis of surfactant positive cells was assessed by a non-parametric Mann–Whitney U test. Generally, the nominal alpha level was set to 5% for statistical analysis in an exploratory manner and p-values of the corresponding F-statistics are presented (*p ˂ 0.05, **p ˂ 0.01, ***p < 0.001 and ****p ˂ 0.0001). Data input, processing, management and analyses were conducted using GraphPad Prism 9.0 (GraphPad Software).

## Supplementary Information

Supplementary Information.
