# Supplementary material for: Functional human iPSC-derived alveolar-like cells cultured in a miniaturized 96‑Transwell air–liquid interface model
Source: Sci Rep. 2021 Aug 23;11:17028. doi: 10.1038/s41598-021-96565-4 (PMC8382767; doi:10.1038/s41598-021-96565-4)
Supplement: Supplementary file 1 — Supplementary Information. [file 41598_2021_96565_MOESM1_ESM.pdf]

# Supplementary Information

## Functional human iPSC-derived alveolar-like cells cultured in a miniaturized 96-Transwell air-liquid interface model

Teresa Bluhmki[1*], Stefanie Traub[2], Ann-Kathrin Müller[2], Sarah Bitzer[1], Eva Schruf[3], Marie-Therese Bammert[3], Marcel Leist[4], Florian Gantner[5], James Garnett[3], Ralf Heilker[1]

Departments of [1]Drug Discovery Sciences, [3]Immunology & Respiratory Diseases Research, Boehringer Ingelheim Pharma GmbH & Co. KG, 88397 Biberach an der Riss, Germany; [2]Trenzyme GmbH, Byk-Gulden–Str. 2, 78467 Konstanz, Germany, [4]In-vitro Toxicology and Biomedicine, University of Konstanz, 78457 Konstanz, Germany, [5]Department of Translational Medicine and Clinical Pharmacology, C. H. Boehringer Sohn AG & Co. KG, 88397 Biberach an der Riss, Germany.

* Corresponding author

teresa.bluhmki@boehringer-ingelheim.com

## Supplementary Figures

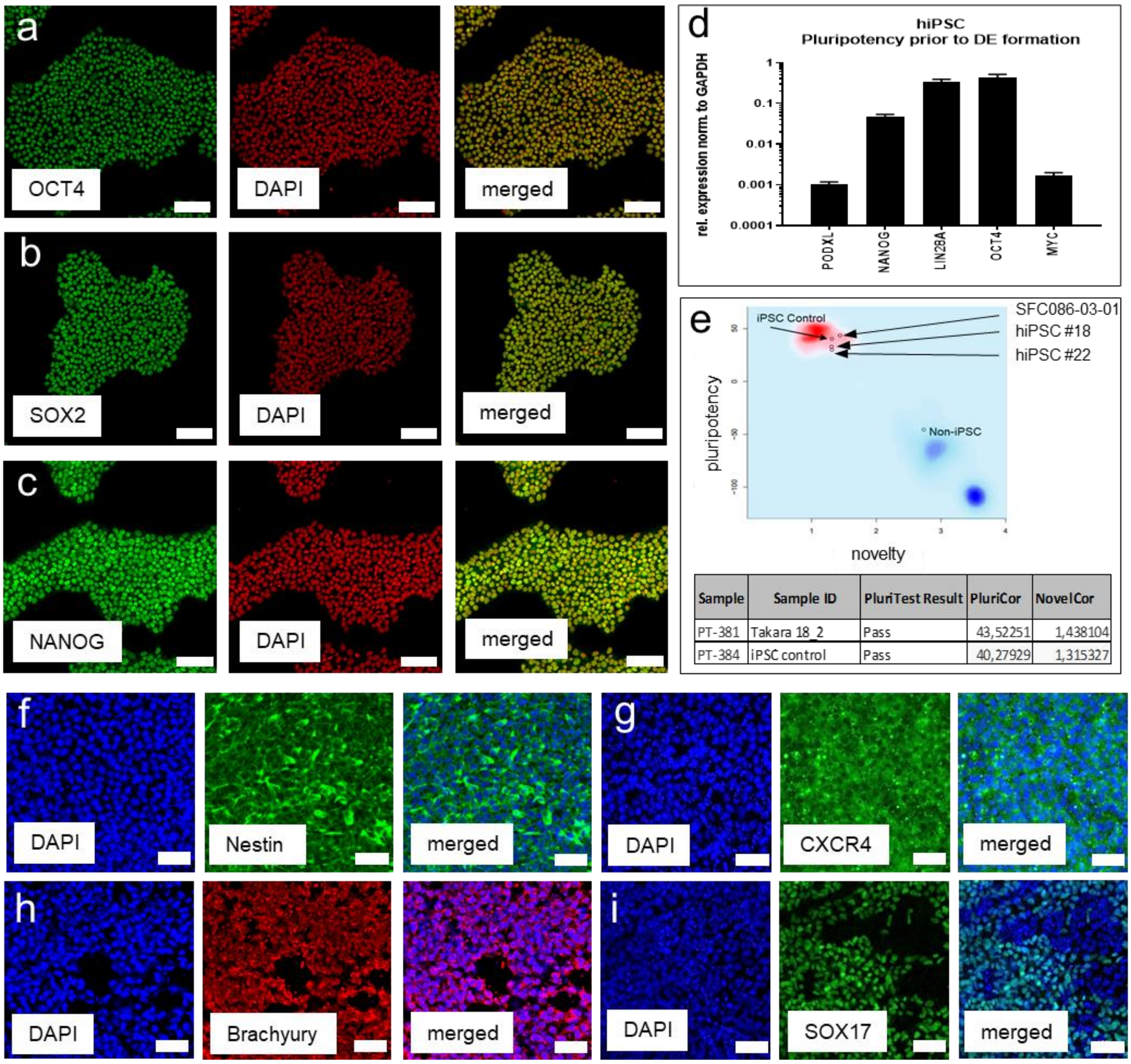


**Figure S 1: Verification of pluripotency and differentiation potential. (a-c)** Immunofluorescence staining of hiPS cell line for classical pluripotency markers. Scale bar = 50 µm **(d)** Verification of pluripotency by RT-PCR**. (e)** Pluripotency and novelty x/y scatter plot showing the comparison of hiPSC to non-hiPS cells. **(f-i)** Confirmation of differentiation into three different germ layers. Immunofluorescence staining of the ectoderm marker Nestin, (f), endoderm marker CXCR4, SOX17 (g+i) and of the mesoderm marker Brachyury (h). Scale bar = 50 µm.

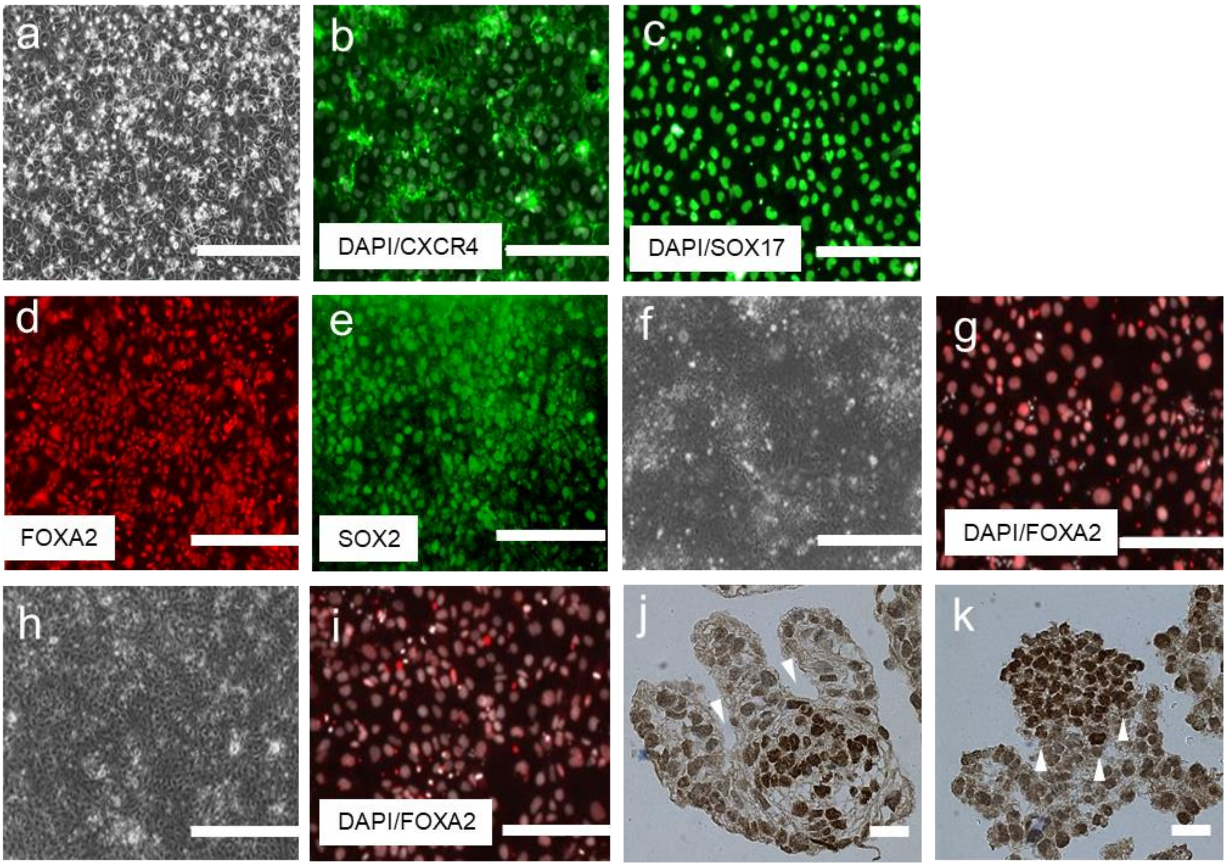


l

| Stage | Seeding next Stage e6 c/cm² | Format [cm²] | Cells total before Freezing e6 c | e6 c/cm² before Freezing | Cells total after Thawing e6 c | e6 c/cm² after Thawing | Thawing Yield [%] | Differentiation Yield [%] |
|---|---|---|---|---|---|---|---|---|
| hiPSC | 0,16 | | | | | | | |
| DE | 0,10 | 300,00 | 85,50 | 0,2850 | 51,30 | 0,1710 | 0,60 | 106,875 |
| LPC | 0,50 | 60,00 | 102,00 | 1,7000 | 45,90 | 0,7650 | 0,45 | 447,368 |
| hiPSC - LPC | | | | | | | | 478,125 |

**Figure S2: Further characterization of cells generated with the optimized differentiation protocol. (a)** Bright field picture of classical morphological coble stone shape of DE cells after thawing. Scale bar = 400 µm **(b+c)** Immunofluorescence staining of hiPSC derived DE cells after thawing for classical DE markers. Scale bar = 200 µm **(d+e)** Verification of maintained differentiation potential of DE cells after the freezing thawing cycle towards the AFE phenotype**.** Scale bar = 200 µm **(f+g)** morphology (f) and FOXA2 positive staining (g) of AFE cells generated with non-cryopreserved DE cells. Scale bar = 200 µm. **(h+i)** morphology (h) and FOXA2 positive staining (i) of AFE cells generated with cryopreserved DE cells. Scale bar = 200 µm**. (j+k)** Budding and branching potential of hiPSC derived lung progenitor cells cultured in 3D AggreWell™ plates from day 21 onwards without switching to a terminal differentiation medium. White arrow heads = bifurcation points. Scale bar = 100 µm. **(l)** Overview of cell yield from hiPSC to LPCs.

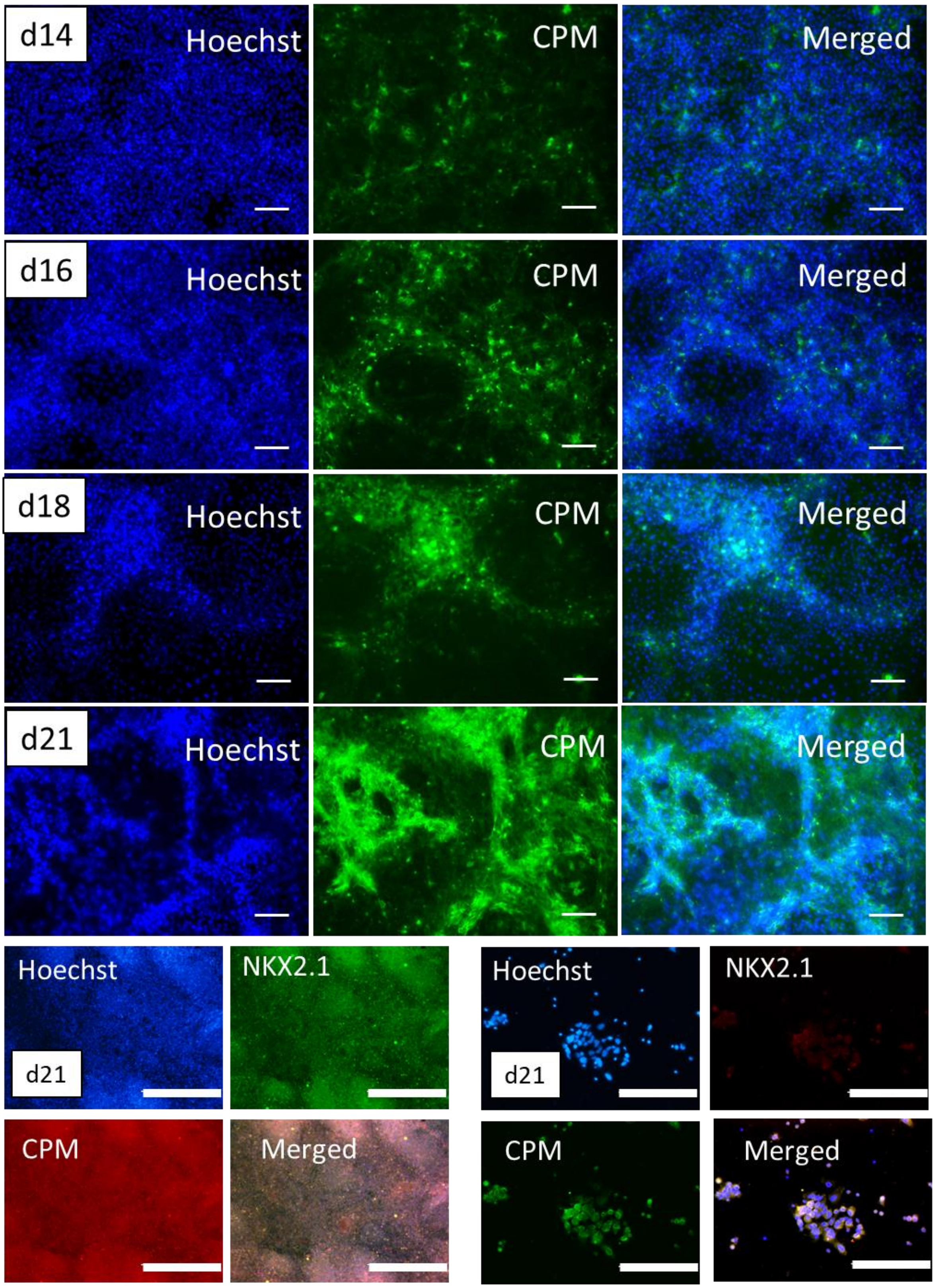


**Figure S3: Further characterization of cells generated out of cryopreserved DE cells.** Increase in CPM expression during vAFE to LP differentiation, a classical lung progenitor surface marker verified by immunofluorescence staining at day 14, day 16, day 18 and day 21 of differentiation. Scale bar = 100 µm. Double staining of NKX2.1 and CPM at day 21 of differentiation. Scale bar =400/ 200µm.

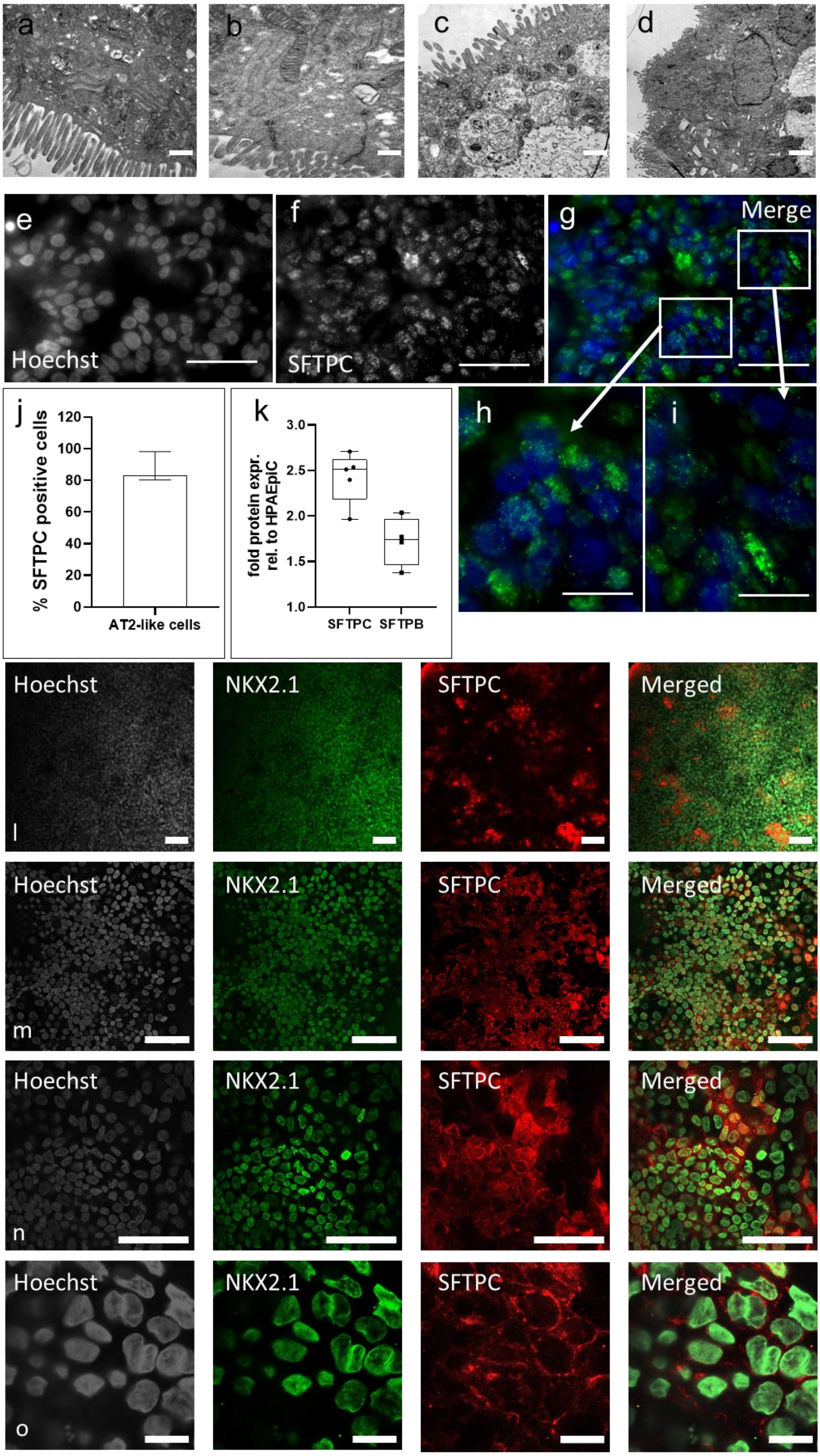


**Figure S4: Additional verifications of alveolar epithelium phenotype of hiPSC derived AT2-like cells. (a-d)** Additional TEM images showing the presence of lamellar bodies and microvilli. Scale bar = 500/ 100nm **(e-i)** Representative pictures of clear intracellular storage of the surfactant protein C (green SFTPC). Scale bar = 100 µm/ 25 µm. **(j)** Semi-quantitative analysis of SFTPC positive cell population in the final hiPSC-derived AT2-like cell ALI cultures (day 27). **(k)** Fold change protein expression of surfactant proteins relative to HPAEpiC in hiPSC-derived AT2-like cells (day 27). **(l-o)** Double staining of NKX2.1 and SFTPC in AT2-like cell ALI cultures (day 27) with 20x, 49x and 60x magnification. Scale bars = 50µm (l-n); 10µm (o)

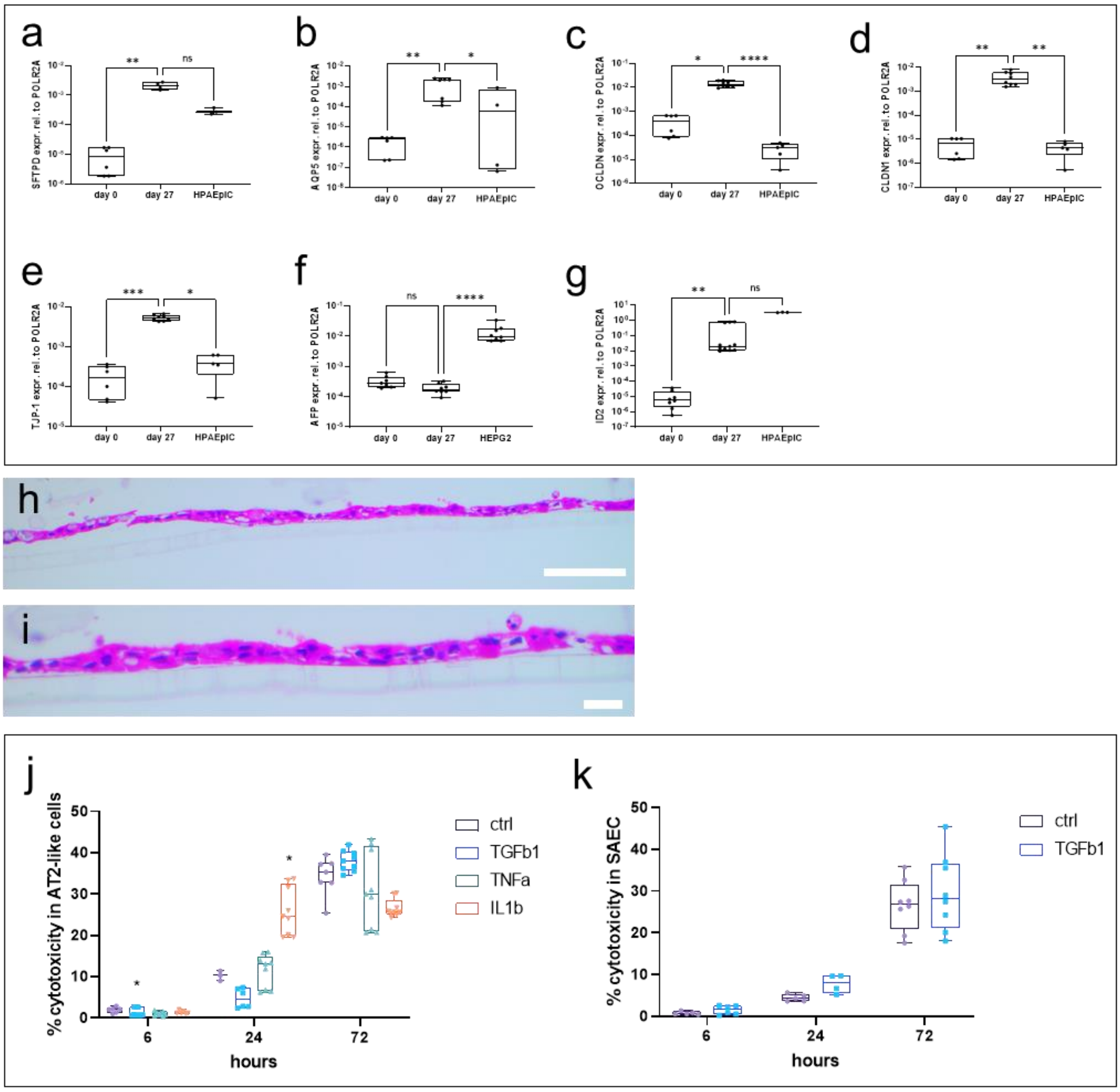


**Figure S5: Further characterization of AT2-like cells. (a-g)** Expression levels of AT2 or hepatocyte marker (AFP) in comparison to Human Pulmonary Alveolar Epithelial Cells (HPAEpiC). **(h-i)** Haematoxylin and Eosin staining of AT2-like cells at day 27 of differentiation. Scale bar = 200 µm (h), 50 µm (i). **(j-k)** Cytotoxicity measurements of different stimulation conditions over the time course of 72 hours in hiPSC-derived cells (j) and human small airway epithelial cells (SAEC) (k).

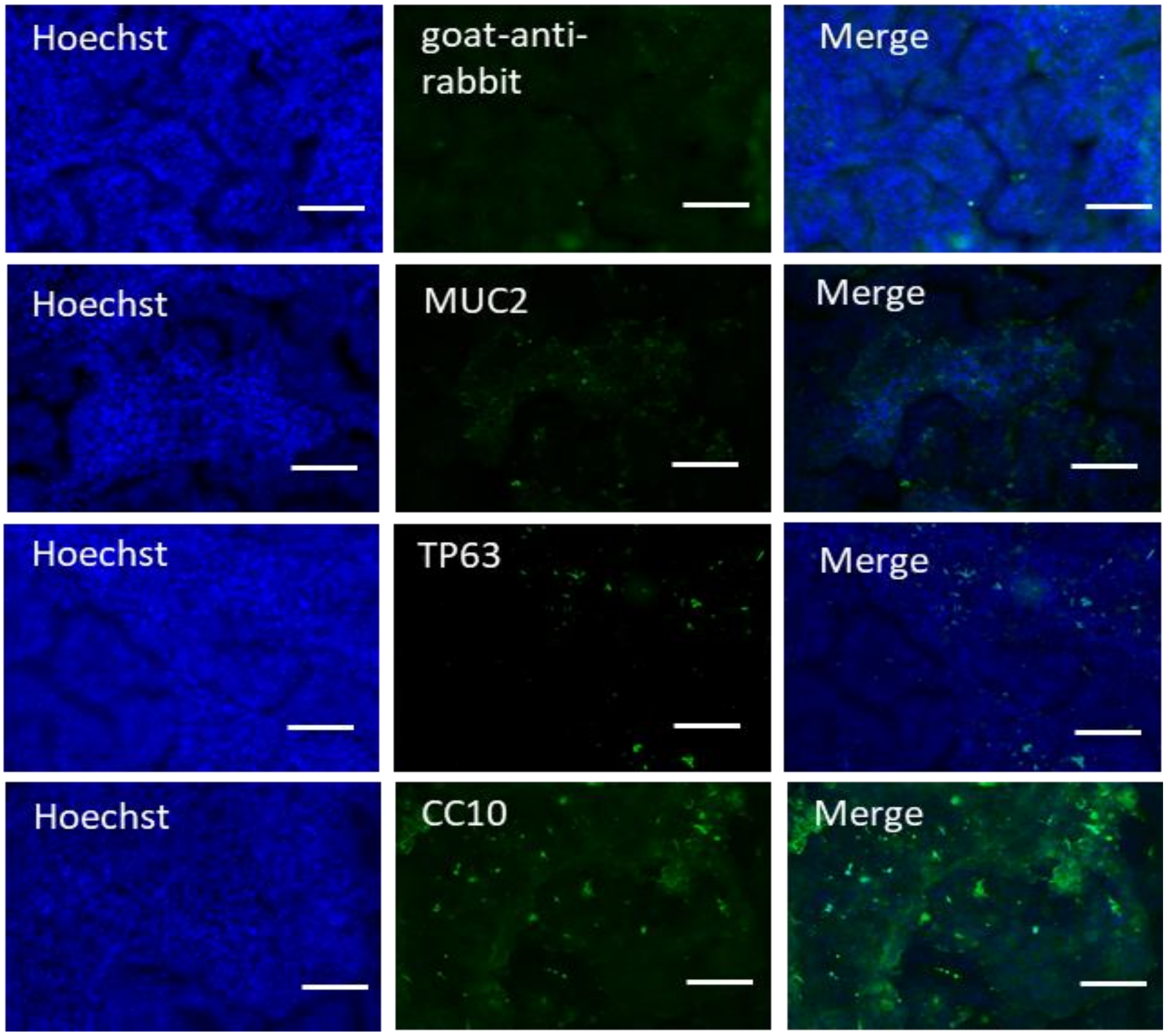


**Figure S6: Evaluation of proximal airway markers in hiPSC derived AT2-like cells.** Immunofluorescence staining of proximal airway markers in AT2-like cells, such as MUC2 (goblet cells), TP63 (basal cells) and CC10 (club cells). Including negative control of secondary antibody (goat anti-rabbit) only. Scale bar = 200 μm.

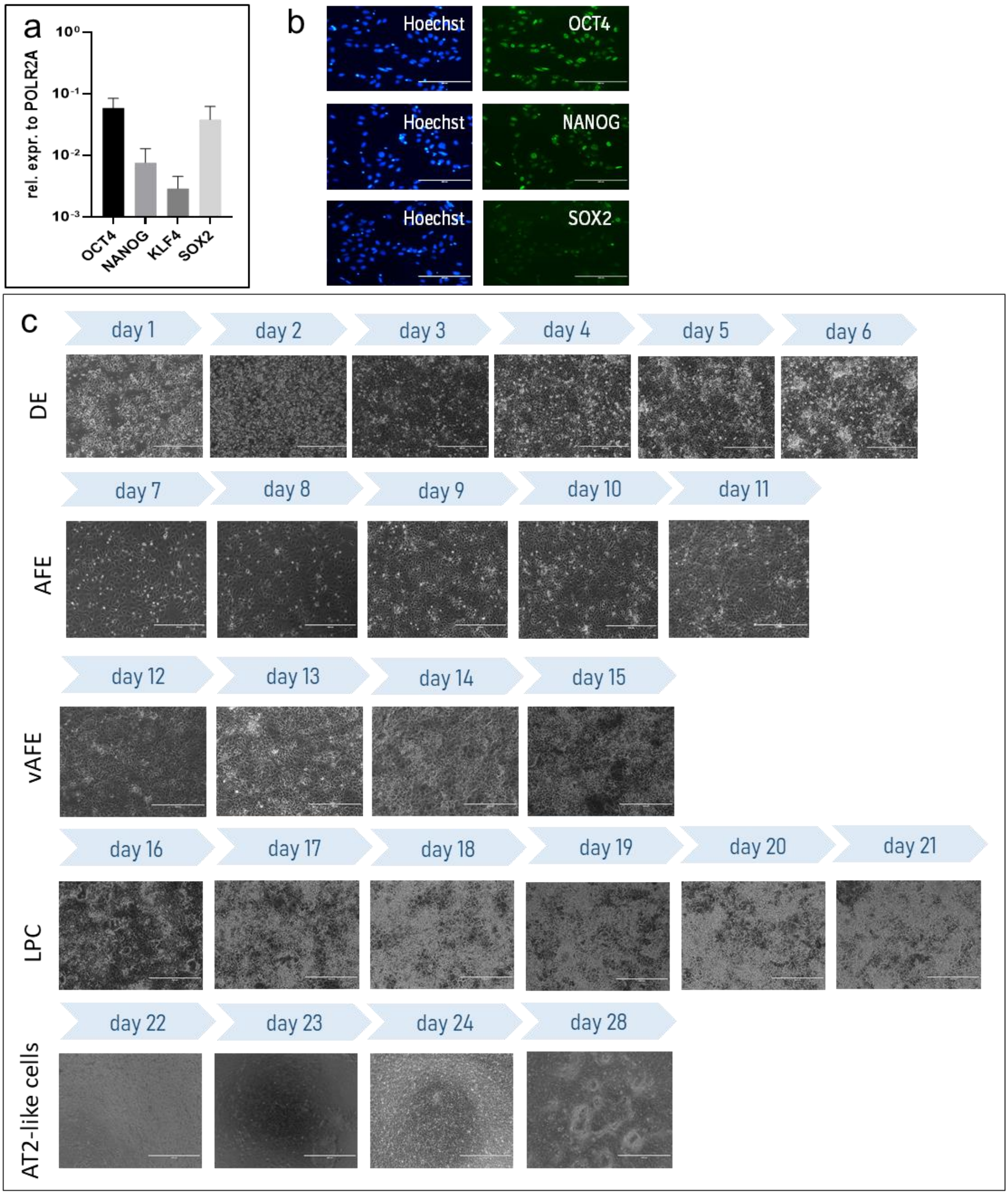


**Figure S7: Characterization of additional hiPSC cell line ChiPSC#22 and verification of its pluripotency. (a)** qRT-PCR analysis of the pluripotency markers OCT4, NANOG, KLF4 and SOX2 **(b)** Immunofluorescence staining of the pluripotency markers OCT4, SOX2 and NANOG in ChiPSC#22. Scale bar = 200 µm. **(c)** Bright field pictures of the whole differentiation process including freezing and thawing steps described in the main text.

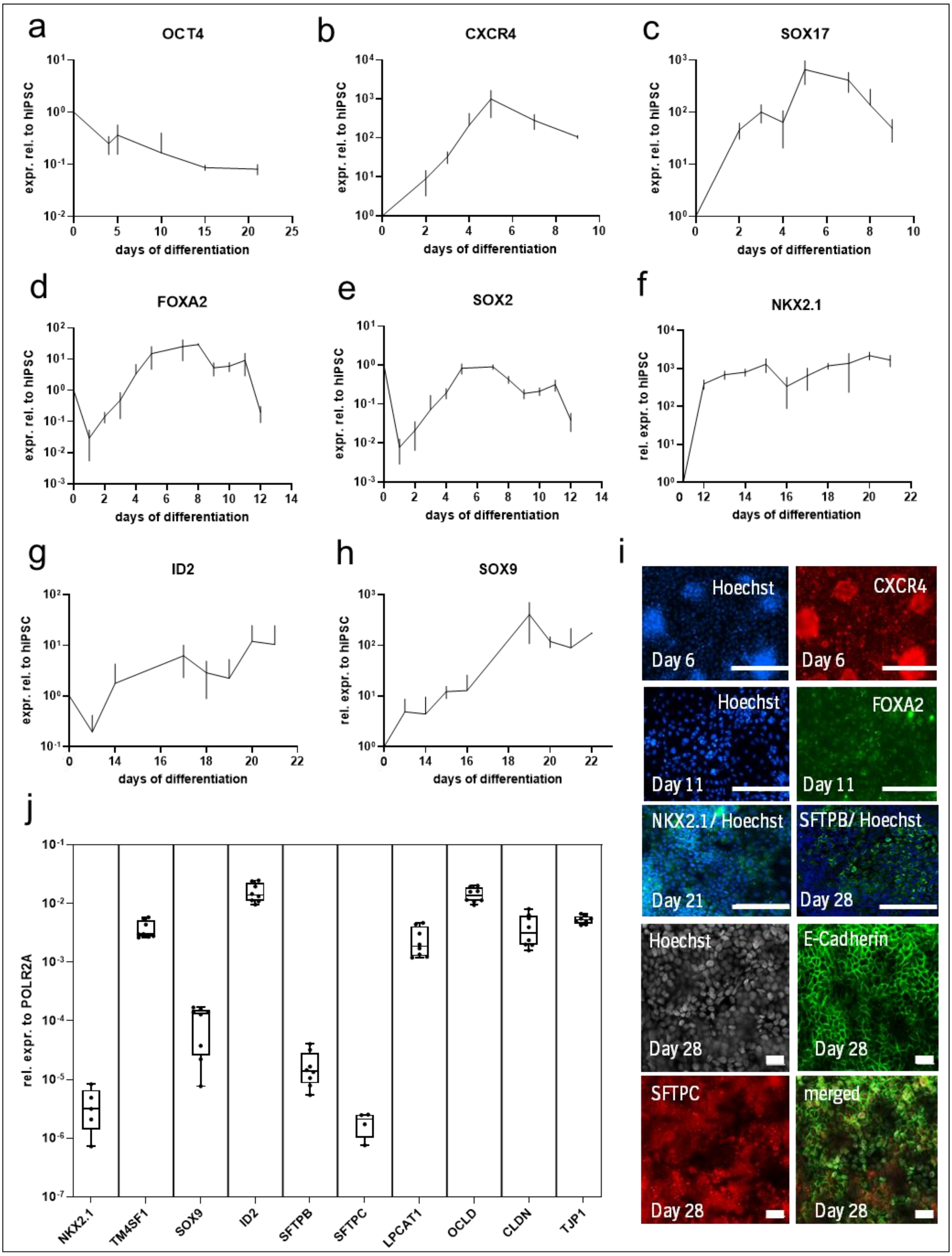


**Figure S8: Confirmation of AT2-like phenotype and robustness of protocol with an alternative hiPSC line; verified by both protein and mRNA level. (a-h)** Relative expression levels of specific differentiation markers, representing different states at daily intervals during differentiation. **(j)** One-step RT-PCR analysis of mature alveolar epithelium cells. **(i)** Immunofluorescence staining of DE marker (CXCR4), AFE marker (FOXA2), LPC marker (NKX2.1) and mature AT2 markers (SFTPC, SFTPB, E-Cadherin). Scale bar = 20 µm/ 200 µm.

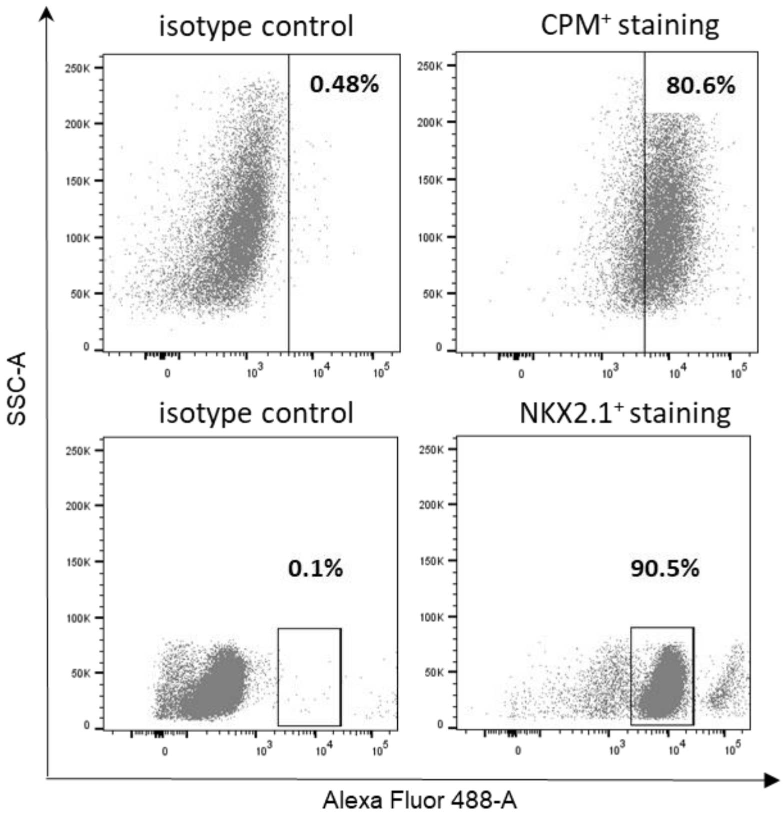


**Figure S9: Flow cytometry analysis of LPCs in alternative hiPSC line ChiPSC22.** Representative FACS plots based on expression of CPM and NKX2.1 (markers for LPCs) in hiPSC-derived LPCs (day 21). Percentages of positive cells are shown in each plot based on isotype control gating strategies.

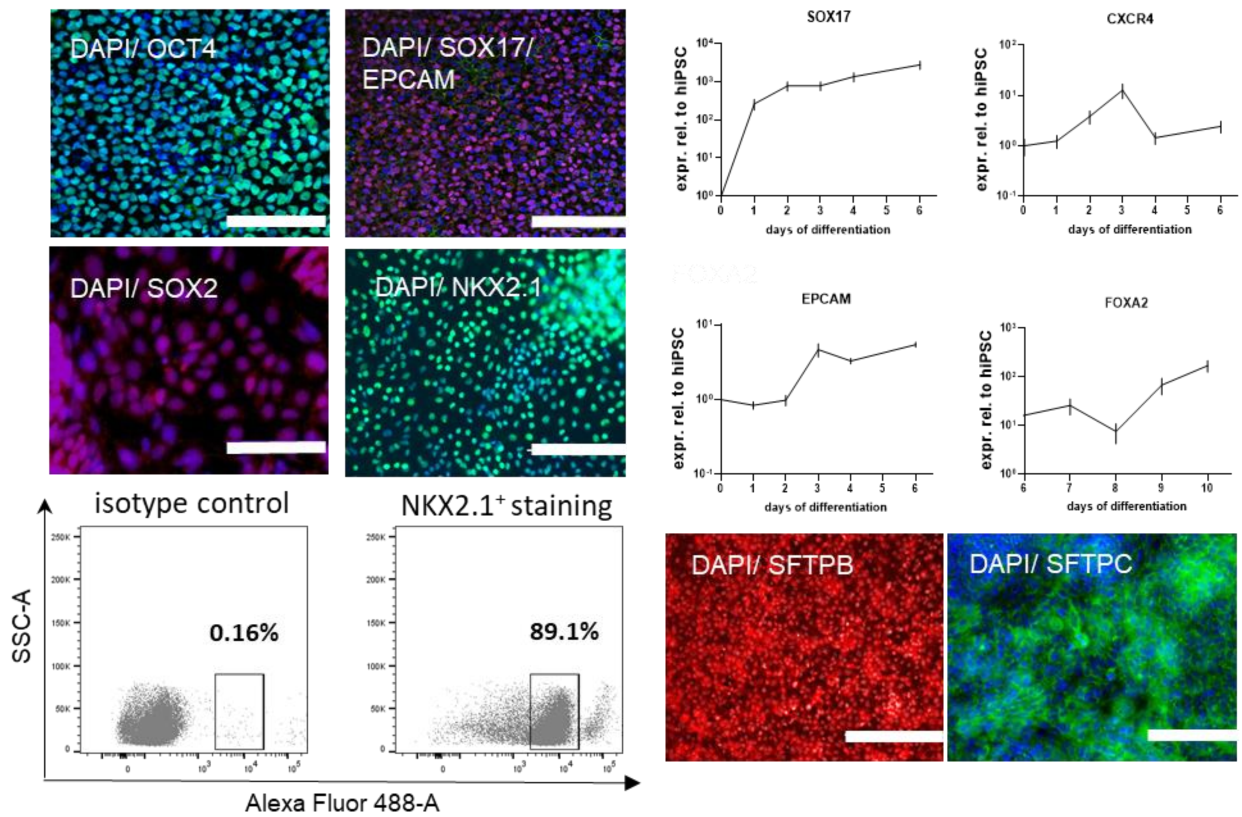


**Figure S10: Characterization and differentiation of a third hiPSC clone SFC-86-03-01.** qRT-PCR analysis of the marker *SOX17, CXCR4, EPCAM and FOXA2.* Immunofluorescence staining of markers for pluripotency OCT4 and differentiation stages, SOX2, NKX2.1 SFTPB, SFTPC. Scale bar = 200/ 100 µm. Flow cytometric analysis of LPC marker NKX2.1 at day 21 of LPCs differentiation. Percentages of positive cells are shown in each plot based on isotype control gating strategies.

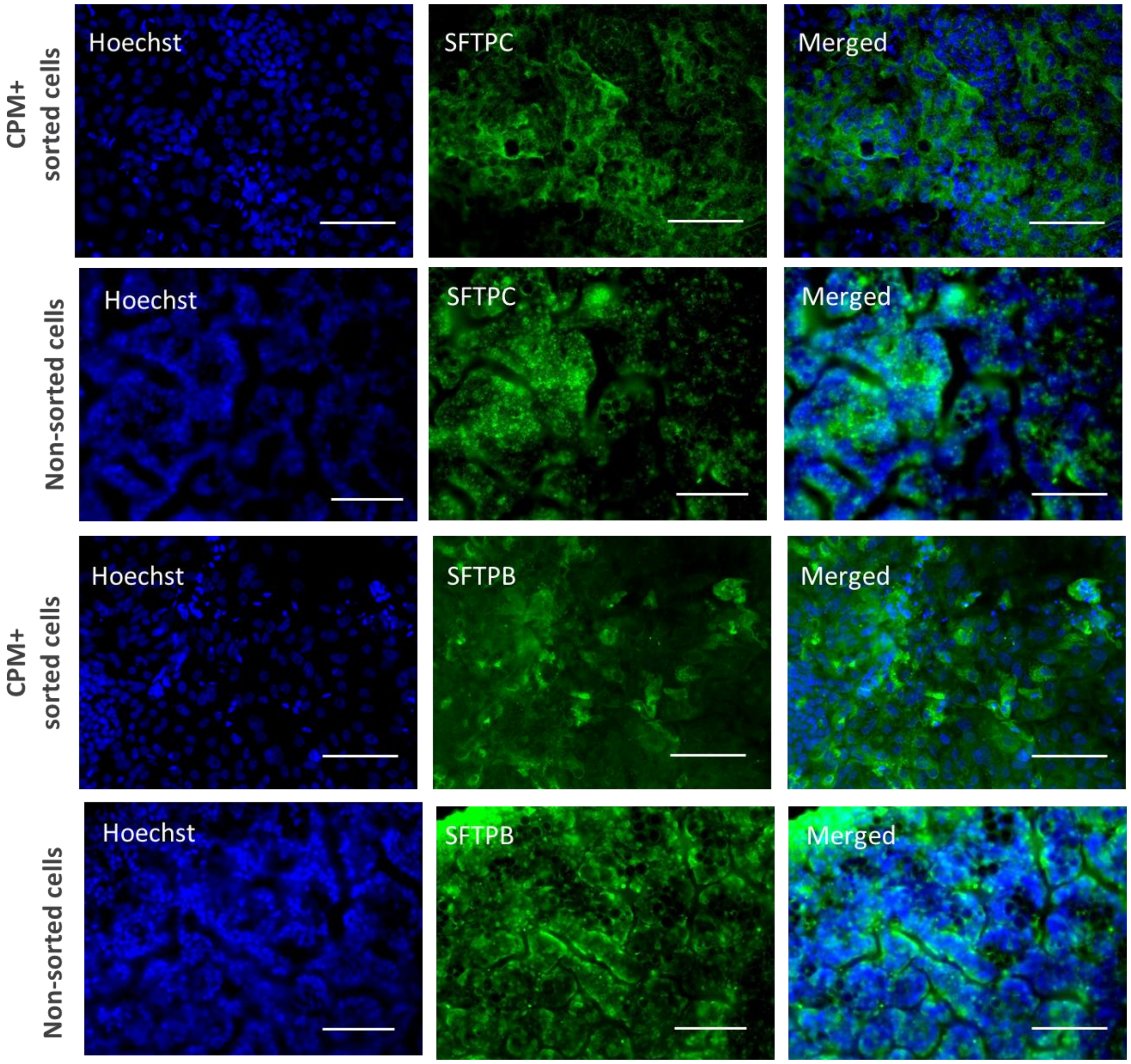


**Figure S11:** Comparison of differentiation efficiency of CPM+ sorted and non-sorted cells, based on the alveolar cell markers SFTPC and SFTPB at day 27 of differentiation. Scale bars = 200µm.

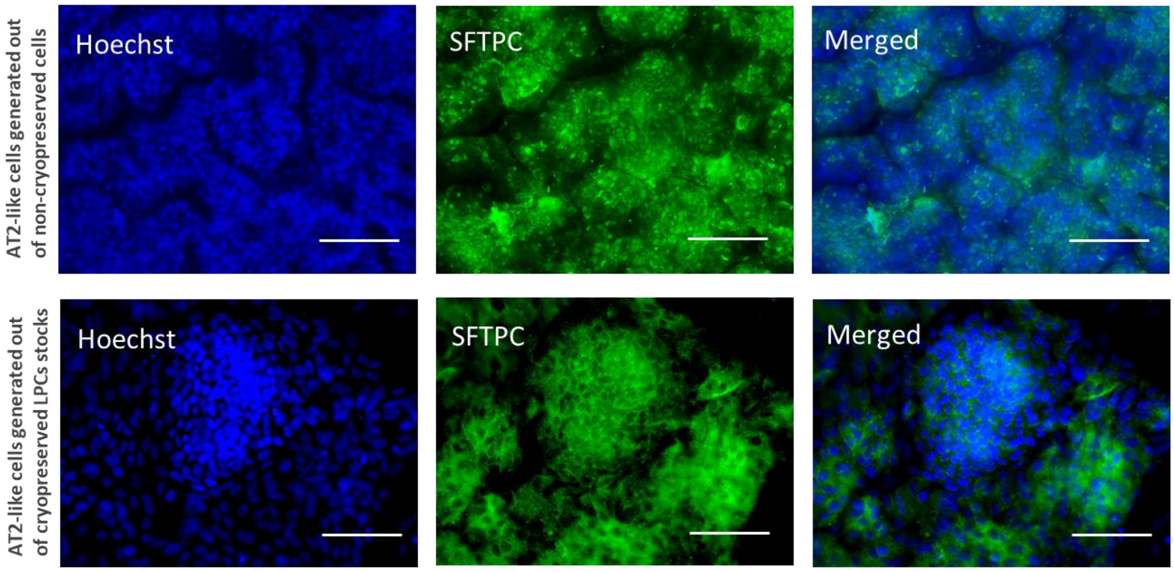


**Figure S12:** Comparison of differentiation efficiency of cryopreserved LPCs sorted and non-cryopreserved LPCs, based on the alveolar cell markers SFTPC at day 27 of differentiation. Scale bars = 200µm

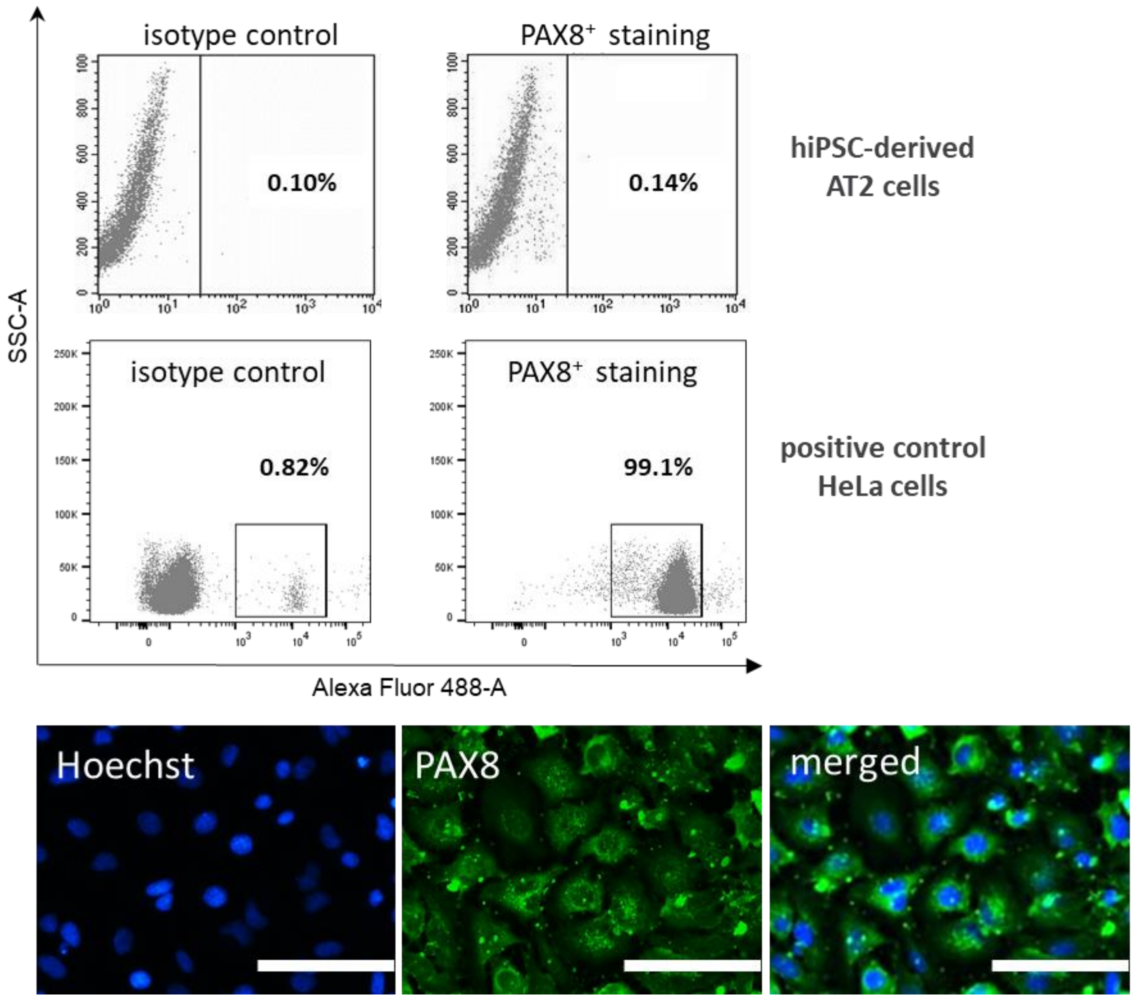


**Figure S13:** Representative FACS plots of hiPSC-derived LPCs regarding PAX8 expression at day 21 of differentiation (marker for thyroid-like cells). Percentages of positive cells are shown in each plot based on isotype control gating strategies. Positive control of PAX8 antibody, verified by flow cytometric analysis and immunofluorescence staining of HeLa cells. Scale bar = 100µm.

## Supplementary Methods

**Pluricard:**

Selected hiPSC cell lines were tested for their pluripotency using PluriTest™ service provided by Life Technologies Corporation. In this assay, the transcriptional profile of the samples is assessed against a reference set of >450 samples including 223 hESC lines, 41 iPSC lines, somatic cells, and tissues to generate a Pluripotency and Novelty Score. Pluripotency score indicates how strongly the samples correlate to the model-based pluripotency matrix. Positive pluripotency scores refer to a highly expressed model-based pluripotency signature. The Novelty Score is an indication of how good the model fits for the respective sample. Low Novelty Scores suggest a good reconstruction of the sample with the PluriTest™ model. For the testing, RNA was isolated from hiPSCs with PureLink™ RNA Mini Kit (#12183025, Thermo Fisher Scientific, Waltham, Massachusetts, USA) according to manufacturer's protocol and quantified using the NanoDrop™. Additionally, contaminating genomic DNA was removed using DNA-free™ Kit (#AM1906, Thermo Fisher Scientific, Waltham, Massachusetts, USA) following the protocol. GeneChip® for PluriTest™ was prepared adding 250 ng total RNA.  Bioinformatical analysis of the samples was performed to authenticate pluripotency potential by generating Pluripotency Score (PluriCor) and Novelty Score (NovelCor) shown in the table. Passing PluriTest™ means significant match with pluripotency matrix, whilst failing is indicative for non-pluripotency. A non-iPSC sample serves as negative control. The scatter plot provides a visualization of the Novelty Score (x-axis) together with the Pluripotency Score (y-axis). Highlighted in red and blue is the empirical distribution of pluripotent (red) and non-pluripotent (blue) samples integrated in the reference data set. A non-iPSC sample serves as negative control.

**Three germ layer formation:**

Three germ layer differentiation was performed according to STEMdiff™ Trilineage Differentiation Assay Kit (#05230, STEMCELL Technologies, Vancouver, Canada). hiPSC were seeded in the recommended density in mTeSR™1 (#85850, STEMCELL Technologies, Vancouver, Canada) with 10 µM Y-27623 (cat. ab120129; Abcam; Cambridge, UK) on 24-well-plates and incubated at standard conditions (37 °C;

5 % $CO_2$). After 24 h, medium was exchanged to the appropriate STEMdiff$^{TM}$ trilineage medium (Ectoderm, Mesoderm, and Endoderm media). The previous step was repeated every day until day 5 for mesoderm and endoderm lineage or day 7 for ectoderm lineage differentiation. After the differentiation time, cells were fixed for visualization of lineage-specific markers via immunofluorescence as previously described.

**H&E transwell staining:**

Transwell insert membranes were fixed with 4 % of Paraformaldehyde (BosterBio, Pleasanton, CA, US) at room temperature for 30 minutes and afterwards removed from transwell tray for further analyzing procedures. Membranes were dehydrated in an ascending series of ethanol and embedded into paraffin according to standard procedures. Paraffin-embedded inserts samples were cut to cross-sections of 3 µm thickness. The samples were rehydrated in a descending series of ethanol and Hematoxylin and eosin (H&E) staining was performed following standard protocols. Images were taken with an AxioCam MR3 (Zeiss, Oberkochen, Germany) using a 20x objective of an Axio Imager Z1 (Zeiss, Oberkochen, Germany).

**Branching of lung progenitor cells:**

Budding and branching potential of hiPSC derived lung progenitor cells was induced in 3D similar to previous reports[1]. To check the feasibility of cultivating LPCs as spheroids, DE cells were differentiated until day 18 and seeded into Aggrewell 400 24-well plates (cat. 34411, Stemcell Technologies; Vancouver, Canada) containing 1200 microwells per 24-well (2000 cells per microwell). Therefore, cells were briefly detached with warm 0.05% Trypsin/ 0.53 mM EDTA and plated into the Aggrewell 400 plate as a single cell suspension. The spheroids kept their morphology up to day 27, with medium change every other day. Subsequent staining procedures are similar to the H&E staining with NKX2.1 antibody and a following DAB-staining according to standard procedures.

**Viability assay:**

Cell viability was verified using LDH-Glo™ Cytotoxicity Assay (#J2380, Promega, Madison, Wisconsin, United States) according to manufacturer's guidelines. Therefore, cell culture supernatant was mixed with LDH standard dilution, incubated for 1 h at room temperature. Maximum LDH release control was generated with Small Airway Epithelilal cells (SAEC). SAEC were stimulated with 2µl 10% Triton X-100 per 100µl for 10–15 minutes or longer before collecting supernatant. Medium background was determined using a no-cell control. Possible cytotoxicity was calculated regarding the following equation:

$$\%\ Cytotoxicity = 100 * \frac{(Experimental\ LDH\ Release - Medium\ Background)}{(Maximum\ LDH\ Release\ Control - Medium\ Background)}$$

Luminescence was recorded being proportional to LDH present in the supernatant by the use of the Safire 2 (Tecan) multi-detection plate reader.

**Digital Western Blot Analysis:**

Digital Simple Western Blotting by Peggy Sue™ for the comparison of protein analysis of hiPSC-derived AT2-like cells and HPAEpiC. The automated digital western blotting platform Peggy Sue™ applies capillary electrophoresis, identifying and quantifying proteins of interest. Protein separation and analysis was performed with a Peggy Sue™ detection system according to the manufacturer's instructions using the 12-230 kDa separation module and the anti-rabbit/ anti-goat detection module, in order to investigate the protein expression of SFTPC and SFTPB in hiPSC-derived AT2-like cells and HPAEpiC, respectively. Cells were lysed with 25 µl/well RIPA lysis buffer. One part of the lysate sample was used, mixed with four parts of fluorescent master mix and was denatured at 95 °C for 5 minutes using a Biometra TProfessional Thermal Cycler. The ladder, denatured samples, blocking reagent (antibody diluent), primary antibodies (1:50 in antibody diluent), HRP-conjugated secondary antibodies, luminol-peroxide mix, stacking and separation matrix were transferred into the plate. Next, the plate was centrifuged for 5 minutes at 1500 g. The following instrument default settings were used: stacking and separation at 250 V for 40 minutes; blocking reagent for 23 minutes; primary and secondary antibody for 30 minutes, respectively; and chemiluminescence detection for 15 minutes. Compass Software v3.1 was used to process and analyze all data results.

# Supplementary Materials

***Table S1*** *Literature based TEER value of primary human alveolar lung cells*

| TEER values (Ωx$cm^2$) | Reference |
|---|---|
| **1000–2000** | Fuchs et al., 2003[2] |
| **1113±30** | Lehmann et al., 2011[3] |
| **>800** | Huh et al., 2010[4] |
| **1250±350** | Hermanns et al., 2009[5] |

***Table S2*** *List of primary antibodies used in this study.*

| Antibody | Cat. No. | Vendor | Dilution |
|---|---|---|---|
| **NKX2.1** | WRAB1231 | Seven Hills | 1:1000 |
| **SFTPB** | WRAB-48604 | Seven Hills | 1:1000 |
| **SFTPC** | WRAB-9337 | Seven Hills | 1:2000 |
| **MUC2** | ab11197 | Abcam | 1:150 |
| **CC10** | ab40873 | Abcam | 1:100 |
| **TP63** | ab735 | Abcam | 1:50 |
| **CD184 (CXCR4)** | 60089 | Stemcell Technologies | 1:50 |
| **SOX17** | AF1924 | R&D Systems | 1:250 |
| **FOXA2** | Af2400 | R&D Systems | 1:50 |
| **SOX2** | #9656 | Cell Signalling | 1:50 |
| **CPM** | 014-27501 | WAKO | 1:200 |
| **CAV1** | ab2910 | Abcam | 1:500 |
| **E-Cadherin** | 13-1700 | Invitrogen | 1.200 |
| **NANOG** | D73G4 | Cell Signalling | 1:1000 |
| **OCT4** | C30A3 | Cell Signalling | 1:1000 |
| **NESTIN** | 60091.1 | Stem Cell Technologies | 1:50 |
| **Brachyury** | 81694 | Cell Signalling | 1:1000 |
| **DAPI** | 4083 | Cell Signalling | 0.1 µg/ml |
| **Hoechst33342** | H3570 | Thermo Fisher | 1:5000 |

***Table S3 List*** *of TaqMan Gene Expression Assays used within this study.*

| Target | Assay ID | Vendor |
|---|---|---|
| **AQP5** | Hs00387048_m1 | Applied Biosystems |
| **AFP** | Hs00173490_m1 | Applied Biosystems |
| **CLDN** | Hs00221623_m1 | Applied Biosystems |
| **CXCR4** | Hs00237052_m1 | Applied Biosystems |
| **FOXA2** | Hs00232764_m1 | Applied Biosystems |
| **ID2** | Hs04187239_m1 | Applied Biosystems |
| **KRT5** | Hs00361185_m1 | Applied Biosystems |
| **LIN28** | Hs00702808_s1 | Applied Biosystems |
| **LPCAT1** | Hs00227357_m1 | Applied Biosystems |

| | | |
|---|---|---|
| **MUC5AC** | Hs01365616_m1 | Applied Biosystems |
| **MUC5B** | Hs00861595_m1 | Applied Biosystems |
| **MYC** | Hs00153408_m1 | Applied Biosystems |
| **NANOG** | Hs02387400_g1 | Applied Biosystems |
| **NKX2.1** | Hs00968940_m1 | Applied Biosystems |
| **OCLDN** | Hs00170162_m1 | Applied Biosystems |
| **PODXL** | Hs01574644_m1 | Applied Biosystems |
| **POLR2A** | Hs00172187_m1 | Applied Biosystems |
| **POU5F1/ OCT4** | Hs00999632_g1 | Applied Biosystems |
| **SCGB1A1** | Hs00171092_m1 | Applied Biosystems |
| **SFTPB** | Hs00167036_m1 | Applied Biosystems |
| **SFTPC** | Hs00161628_m1 | Applied Biosystems |
| **SFTPD** | Hs01108490_m1 | Applied Biosystems |
| **SOX17** | Hs00751752_s1 | Applied Biosystems |
| **SOX2** | Hs01053049_s1 | Applied Biosystems |
| **SOX9** | Hs00165814_m1 | Applied Biosystems |
| **TJP1** | Hs01551861_m1 | Applied Biosystems |
| **TM4SF1** | Hs01547334_m1 | Applied Biosystems |

***Table S4*** *List of Western Blot reagents and equipment used within this study.*

| **Product** | **content** | **cat. number** | **manufacturer** |
|---|---|---|---|
| 12-230 kDa Separation Module | 96 capillaries, plates, wash buffer, upper and lower running buffers 2, cups, cleaning sponges, separation matrix 2, stacking matrix 2, 10X sample buffer, standard pack 1 with 8X DTT, 8X FL master mix with 26 kDa system control protein | SM-S001 | ProteinSimple |
| Anti-Rabbit Detection Module | luminol-S, peroxide, antibody diluent 2, anti-rabbit secondary antibody, Streptavid-HRP | DM-001 | ProteinSimple |
| Anti-Goat Detection Module | luminol-S, peroxide, antibody diluent 2, anti-goat secondary antibody, Streptavid-HRP | DM-003 | ProteinSimple |
| anti-SFTPC | 1:500 | WRAB-9337 | Seven Hills |
| anti-SFTPB | 1:500 | AB3430 | Millipore |
| anti-GAPDH | 1:100 | AF5718 | R&D Systems |
| Sally Sue™ Detection System with Compass Software v3.1 | | 004-700 | ProteinSimple |
| Heraeus Multifuge X1 Centrifuge | | 75004210 | Thermo Fisher Scientific |
| Biometra TProfessional Thermal Cycler | | 070-901 | Analytik jena |

***Table S5*** *List of total RNA and total cell lysates of HPAEpiC used for comparison studies.*

| Product | content | cat. number | manufacturer |
|---|---|---|---|
| Human Pulmonary Alveolar Epithelial Cell Total RNA | Human Pulmonary Alveolar Epithelial Cell total RNA is prepared from early passage Human Pulmonary Alveolar Epithelial Cells using the Qiagen AllPrep DNA/RNA Mini kit. | 3205-SC | BioTrend |
| Human Pulmonary Alveolar Epithelial Cell Lysate | HPAEpiC Lysate is prepared from early passage Human Pulmonary Alveolar Epithelial Cells using RIPA buffer | 3206 | ScienCell Research Laboratories |

# Supplementary Tables

***Table S4*** *Statistical details for the comparison of day 27 vs. day 0 and HPAEpiC for the NKX2.1 gene expression levels.*

| | | | | | | |
|---|---|---|---|---|---|---|
| Number of families | 1 | | | | | |
| Number of comparisons per family | 2 | | | | | |
| Alpha | 0,05 | | | | | |
| Uncorrected Dunn's test | Mean rank diff, | Significant? | Summary | Individual P Value | B-? | |
| day 27 vs. day 0 | 10,00 | Yes | ** | 0,0039 | A | day 0 |
| day 27 vs. HPAEpiC | 0,000 | No | ns | >0,9999 | C | HPAEpiC |
| Test details | Mean rank 1 | Mean rank 2 | Mean rank diff, | n1 | n2 | Z |
| day 27 vs. day 0 | 14,00 | 4,000 | 10,00 | 5 | 7 | 2,888 |
| day 27 vs. HPAEpiC | 14,00 | 14,00 | 0,000 | 5 | 8 | 0,000 |

***Table S5*** *Statistical details for the comparison of day 27 vs. day 0 and HPAEpiC for the SFTPC gene expression levels.*

| | | | | | | |
|---|---|---|---|---|---|---|
| Number of families | 1 | | | | | |
| Number of comparisons per family | 2 | | | | | |
| Alpha | 0,05 | | | | | |
| Uncorrected Dunn's test | Mean rank diff, | Significant? | Summary | Individual P Value | B-? | |
| day 27 vs. day 0 | 11,00 | Yes | *** | 0,0003 | A | day 0 |
| day 27 vs. HPAEpiC | 6,000 | Yes | * | 0,0290 | C | HPAEpiC |
| Test details | Mean rank 1 | Mean rank 2 | Mean rank diff, | n1 | n2 | Z |
| day 27 vs. day 0 | 13,50 | 2,500 | 11,00 | 6 | 4 | 3,579 |
| day 27 vs. HPAEpiC | 13,50 | 7,500 | 6,000 | 6 | 6 | 2,183 |

***Table S6*** *Statistical details for the comparison of day 27 vs. day 0 and HPAEpiC for the LPCAT1 gene expression levels.*

| | | | | | | |
|---|---|---|---|---|---|---|
| Number of families | 1 | | | | | |
| Number of comparisons per family | 2 | | | | | |
| Alpha | 0,05 | | | | | |
| Uncorrected Dunn's test | Mean rank diff, | Significant? | Summary | Individual P Value | B-? | |
| day 27 vs. day 0 | 10,17 | Yes | ** | 0,0029 | A | day 0 |
| day 27 vs. HPAEpiC | 9,458 | Yes | ** | 0,0031 | C | HPAEpiC |
| Test details | Mean rank 1 | Mean rank 2 | Mean rank diff, | n1 | n2 | Z |
| day 27 vs. day 0 | 17,33 | 7,167 | 10,17 | 6 | 6 | 2,976 |
| day 27 vs. HPAEpiC | 17,33 | 7,875 | 9,458 | 6 | 8 | 2,960 |

***Table S7*** *Statistical details for the comparison of day 27 vs. day 0 and HPAEpiC for the SFTPB gene expression levels.*

| | | | | | | |
|---|---|---|---|---|---|---|
| Number of families | 1 | | | | | |
| Number of comparisons per family | 2 | | | | | |
| Alpha | 0,05 | | | | | |
| Uncorrected Dunn's test | Mean rank diff, | Significant? | Summary | Individual P Value | B-? | |
| day 27 vs. day 0 | 13,94 | Yes | **** | <0,0001 | A | day 0 |
| day 27 vs. HPAEpiC | 3,302 | No | ns | 0,3723 | C | HPAEpiC |
| Test details | Mean rank 1 | Mean rank 2 | Mean rank diff, | n1 | n2 | Z |
| day 27 vs. day 0 | 18,94 | 5,000 | 13,94 | 9 | 9 | 4,029 |
| day 27 vs. HPAEpiC | 18,94 | 15,64 | 3,302 | 9 | 7 | 0,8922 |

***Table S8*** *Statistical details for the comparison of day 27 vs. day 0 and HPAEpiC for the ABCA3 gene expression levels.*

| | | | | | | |
|---|---|---|---|---|---|---|
| Number of families | 1 | | | | | |
| Number of comparisons per family | 2 | | | | | |
| Alpha | 0,05 | | | | | |
| Uncorrected Dunn's test | Mean rank diff, | Significant? | Summary | Individual P Value | B-? | |
| day 27 vs. day 0 | 7,500 | Yes | ** | 0,0033 | A | day 0 |
| day 27 vs. HPAEpiC | 3,000 | No | ns | 0,2393 | C | HPAEpiC |
| Test details | Mean rank 1 | Mean rank 2 | Mean rank diff, | n1 | n2 | Z |
| day 27 vs. day 0 | 10,00 | 2,500 | 7,500 | 4 | 4 | 2,942 |
| day 27 vs. HPAEpiC | 10,00 | 7,000 | 3,000 | 4 | 4 | 1,177 |

***Table S9*** *Statistical details for the comparison of day 27 vs. day 0 and HPAEpiC for the CAV1 gene expression levels.*

| | | | | | | |
|---|---|---|---|---|---|---|
| Number of families | 1 | | | | | |
| Number of comparisons per family | 2 | | | | | |
| Alpha | 0,05 | | | | | |
| Uncorrected Dunn's test | Mean rank diff, | Significant? | Summary | Individual P Value | B-? | |
| day 27 vs. day 0 | -13,00 | Yes | **** | <0,0001 | A | day 0 |
| day 27 vs. HPAEpiC | -6,000 | No | ns | 0,0790 | C | HPAEpiC |
| Test details | Mean rank 1 | Mean rank 2 | Mean rank diff, | n1 | n2 | Z |
| day 27 vs. day 0 | 3,500 | 16,50 | -13,00 | 6 | 8 | 4,069 |
| day 27 vs. HPAEpiC | 3,500 | 9,500 | -6,000 | 6 | 6 | 1,757 |

***Table S10*** *Statistical details for the comparison of day 27 vs. day 0 and HPAEpiC for the PDPN gene expression levels.*

| | | | | | | |
|---|---|---|---|---|---|---|
| Number of families | 1 | | | | | |
| Number of comparisons per family | 2 | | | | | |
| Alpha | 0,05 | | | | | |
| Uncorrected Dunn's test | Mean rank diff, | Significant? | Summary | Individual P Value | B-? | |
| day 27 vs. day 0 | -10,00 | Yes | ** | 0,0019 | A | day 0 |
| day 27 vs. HPAEpiC | 4,500 | No | ns | 0,2258 | C | HPAEpiC |
| Test details | Mean rank 1 | Mean rank 2 | Mean rank diff, | n1 | n2 | Z |
| day 27 vs. day 0 | 9,500 | 19,50 | -10,00 | 10 | 8 | 3,108 |
| day 27 vs. HPAEpiC | 9,500 | 5,000 | 4,500 | 10 | 5 | 1,211 |

***Table S11*** *Statistical details for the comparison of day 27 vs. day 0 and HPAEpiC for the CDH1 gene expression levels.*

| | |
|---|---|
| Number of families | 1 |

| | | | | | | |
|---|---|---|---|---|---|---|
| Number of comparisons per family | 2 | | | | | |
| Alpha | 0,05 | | | | | |
| Uncorrected Dunn's test | Mean rank diff, | Significant? | Summary | Individual P Value | B-? | |
| day 27 vs. day 0 | 16,00 | Yes | **** | <0,0001 | A | day 0 |
| day 27 vs. HPAEpiC | 8,000 | Yes | * | 0,0237 | C | HPAEpiC |
| Test details | Mean rank 1 | Mean rank 2 | Mean rank diff, | n1 | n2 | Z |
| day 27 vs. day 0 | 20,50 | 4,500 | 16,00 | 8 | 8 | 4,525 |
| day 27 vs. HPAEpiC | 20,50 | 12,50 | 8,000 | 8 | 8 | 2,263 |

***Table S12*** *Statistical details for the comparison of day 27 vs. day 0 and HPAEpiC for the OCT4 gene expression levels.*

| | | | | | | |
|---|---|---|---|---|---|---|
| Number of families | 1 | | | | | |
| Number of comparisons per family | 2 | | | | | |
| Alpha | 0,05 | | | | | |
| Dunn's multiple comparisons test | Mean rank diff, | Significant? | Summary | Adjusted P Value | B-? | |
| day 27 vs. day 0 | -13,50 | Yes | **** | <0,0001 | A | day 0 |
| day 27 vs. HPAEpiC | -6,000 | No | ns | 0,2969 | C | HPAEpiC |
| Test details | Mean rank 1 | Mean rank 2 | Mean rank diff, | n1 | n2 | Z |
| day 27 vs. day 0 | 4,500 | 18,00 | -13,50 | 8 | 11 | 4,285 |
| day 27 vs. HPAEpiC | 4,500 | 10,50 | -6,000 | 8 | 4 | 1,445 |

***Table S13*** *Statistical details for the comparison of day 27 vs. day 0 and HPAEpiC for the SOX9 gene expression levels.*

| | | | | | | |
|---|---|---|---|---|---|---|
| Number of families | 1 | | | | | |
| Number of comparisons per family | 2 | | | | | |
| Alpha | 0,05 | | | | | |
| Uncorrected Dunn's test | Mean rank diff, | Significant? | Summary | Individual P Value | B-? | |
| day 27 vs. day 0 | 9,650 | Yes | ** | 0,0029 | A | day 0 |
| day 27 vs. HPAEpiC | -0,5000 | No | ns | 0,8936 | C | HPAEpiC |
| Test details | Mean rank 1 | Mean rank 2 | Mean rank diff, | n1 | n2 | Z |
| day 27 vs. day 0 | 15,20 | 5,550 | 9,650 | 5 | 10 | 2,980 |
| day 27 vs. HPAEpiC | 15,20 | 15,70 | -0,5000 | 5 | 5 | 0,1337 |

***Table S14*** *Statistical details for the comparison of day 27 vs. day 0 and HPAEpiC for the SOX2 gene expression levels.*

| | | | | | | |
|---|---|---|---|---|---|---|
| Number of families | 1 | | | | | |
| Number of comparisons per family | 2 | | | | | |
| Alpha | 0,05 | | | | | |
| Uncorrected Dunn's test | Mean rank diff, | Significant? | Summary | Individual P Value | B-? | |
| day 27 vs. day 0 | -11,50 | Yes | *** | 0,0009 | A | day 0 |
| day 27 vs. HPAEpiC | 9,500 | Yes | * | 0,0334 | C | HPAEpiC |
| Test details | Mean rank 1 | Mean rank 2 | Mean rank diff, | n1 | n2 | Z |
| day 27 vs. day 0 | 12,00 | 23,50 | -11,50 | 15 | 8 | 3,310 |
| day 27 vs. HPAEpiC | 12,00 | 2,500 | 9,500 | 15 | 4 | 2,128 |

***Table S15*** *Statistical details for the comparison of day 27 vs. day 0 and HPAEpiC for the TM4SF1 gene expression levels.*

| | |
|---|---|
| Table Analyzed | *TM4SF1 |
| Column C | HPAEpiC |
| vs. | vs, |
| Column B | day 27 |
| Mann Whitney test | |
| P value | 0,4606 |
| Exact or approximate P value? | Exact |
| P value summary | ns |

| | |
|---|---|
| Significantly different (P < 0.05)? | No |
| One- or two-tailed P value? | Two-tailed |
| Sum of ranks in column B,C | 57 , 21 |
| Mann-Whitney U | 11 |
| Difference between medians | |
| Median of column B | 0,003000, n=8 |
| Median of column C | 0,002997, n=4 |
| Difference: Actual | -2,895e-006 |
| Difference: Hodges-Lehmann | -0,0009293 |

| | |
|---|---|
| Theoretical median | 0,000 |
| Actual median | 0,003000 |
| Number of values | 8 |
| Wilcoxon Signed Rank Test | |
| Sum of signed ranks (W) | 36,00 |
| Sum of positive ranks | 36,00 |
| Sum of negative ranks | 0,000 |
| P value (two tailed) | 0,0078 |
| Exact or estimate? | Exact |
| P value summary | ** |
| Significant (alpha=0.05)? | Yes |
| How big is the discrepancy? | |
| Discrepancy | 0,003000 |

***Table S16** Statistical details for the TEER kinetic over the time course of 7 days.*

| | | | | | | |
|---|---|---|---|---|---|---|
| Number of families | 1 | | | | | |
| Number of comparisons per family | 3 | | | | | |
| Alpha | 0,05 | | | | | |
| Uncorrected Dunn's test | Mean rank diff, | Significant? | Summary | Individual P Value | A-? | |
| d1 vs. d2 | 7,360 | No | ns | 0,4548 | B | d2 |
| d1 vs. d3 | -16,33 | No | ns | 0,1707 | C | d3 |
| d1 vs. d7 | -35,83 | Yes | *** | 0,0003 | D | d7 |
| Test details | Mean rank 1 | Mean rank 2 | Mean rank diff, | n1 | n2 | Z |
| d1 vs. d2 | 25,17 | 17,81 | 7,360 | 6 | 31 | 0,7475 |
| d1 vs. d3 | 25,17 | 41,50 | -16,33 | 6 | 8 | 1,370 |
| d1 vs. d7 | 25,17 | 61,00 | -35,83 | 6 | 31 | 3,639 |

***Table S17** Statistical details for the comparison of AT2-like cells, SAEC and HPAEpiC.*

| | | | | | | |
|---|---|---|---|---|---|---|
| Number of families | 1 | | | | | |
| Number of comparisons per family | 3 | | | | | |
| Alpha | 0,05 | | | | | |
| Uncorrected Dunn's test | Mean rank diff, | Significant? | Summary | Individual P Value | | |
| ALIEN vs. HPAEpiC | -3,417 | No | ns | 0,7345 | A-B | |
| ALIEN vs. SAEC | 45,25 | Yes | **** | <0,0001 | A-C | |
| HPAEpiC vs. SAEC | 48,67 | Yes | **** | <0,0001 | B-C | |
| Test details | Mean rank 1 | Mean rank 2 | Mean rank diff, | n1 | n2 | Z |
| ALIEN vs. HPAEpiC | 71,25 | 74,67 | -3,417 | 32 | 9 | 0,3391 |
| ALIEN vs. SAEC | 71,25 | 26,00 | 45,25 | 32 | 51 | 7,515 |
| HPAEpiC vs. SAEC | 74,67 | 26,00 | 48,67 | 9 | 51 | 5,041 |

***Table S18** Statistical details for the physiological changes in the secretory levels of MMP10 by TGFβ-1 stimulation.*

| | | | | | | |
|---|---|---|---|---|---|---|
| Number of families | 1 | | | | | |
| Number of comparisons per family | 3 | | | | | |
| Alpha | 0,05 | | | | | |
| Uncorrected Dunn's test | Mean rank diff, | Significant? | Summary | Individual P Value | | |
| 6h vs. 24h | -5,889 | No | ns | 0,1155 | A-B | |
| 6h vs. 72h | -16,44 | Yes | **** | <0,0001 | A-C | |
| 24h vs. 72h | -10,56 | Yes | ** | 0,0048 | B-C | |
| Test details | Mean rank 1 | Mean rank 2 | Mean rank diff, | n1 | n2 | Z |
| 6h vs. 24h | 6,556 | 12,44 | -5,889 | 9 | 9 | 1,574 |
| 6h vs. 72h | 6,556 | 23,00 | -16,44 | 9 | 9 | 4,395 |
| 24h vs. 72h | 12,44 | 23,00 | -10,56 | 9 | 9 | 2,821 |

***Table S19** Statistical details for the physiological changes in the expression levels of MMP10 by TGFβ-1 stimulation.*

| | |
|---|---|
| Number of families | 1 |
| Number of comparisons per family | 3 |
| Alpha | 0,05 |

| | | | | | | |
|---|---|---|---|---|---|---|
| Uncorrected Dunn's test | Mean rank diff, | Significant? | Summary | Individual P Value | | |
| 6h vs. 24h | -6,000 | No | ns | 0,0516 | A-B | |
| 6h vs. 72h | -12,00 | Yes | **** | <0,0001 | A-C | |
| 24h vs. 72h | -6,000 | No | ns | 0,0516 | B-C | |
| Test details | Mean rank 1 | Mean rank 2 | Mean rank diff, | n1 | n2 | Z |
| 6h vs. 24h | 3,500 | 9,500 | -6,000 | 6 | 6 | 1,947 |
| 6h vs. 72h | 3,500 | 15,50 | -12,00 | 6 | 6 | 3,893 |
| 24h vs. 72h | 9,500 | 15,50 | -6,000 | 6 | 6 | 1,947 |

***Table S20*** *Statistical details for the physiological changes in the secretion levels of Fibronectin by TGFβ-1 stimulation.*

| | | | | | | |
|---|---|---|---|---|---|---|
| Number of families | 1 | | | | | |
| Number of comparisons per family | 3 | | | | | |
| Alpha | 0,05 | | | | | |
| Uncorrected Dunn's test | Mean rank diff, | Significant? | Summary | Individual P Value | | |
| 6h vs. 24h | -9,000 | Yes | * | 0,0161 | A-B | |
| 6h vs. 72h | -18,00 | Yes | **** | <0,0001 | A-C | |
| 24h vs. 72h | -9,000 | Yes | * | 0,0161 | B-C | |
| Test details | Mean rank 1 | Mean rank 2 | Mean rank diff, | n1 | n2 | Z |
| 6h vs. 24h | 5,000 | 14,00 | -9,000 | 9 | 9 | 2,406 |
| 6h vs. 72h | 5,000 | 23,00 | -18,00 | 9 | 9 | 4,811 |
| 24h vs. 72h | 14,00 | 23,00 | -9,000 | 9 | 9 | 2,406 |

***Table S21*** *Statistical details for the physiological changes in the expression levels of Fibronectin by TGFβ-1 stimulation.*

| | | | | | | |
|---|---|---|---|---|---|---|
| Number of families | 1 | | | | | |
| Number of comparisons per family | 3 | | | | | |
| Alpha | 0,05 | | | | | |
| Uncorrected Dunn's test | Mean rank diff, | Significant? | Summary | Individual P Value | | |
| 6h vs. 24h | -6,167 | Yes | * | 0,0248 | A-B | |
| 6h vs. 72h | -10,75 | Yes | *** | 0,0005 | A-C | |
| 24h vs. 72h | -4,583 | No | ns | 0,1356 | B-C | |
| Test details | Mean rank 1 | Mean rank 2 | Mean rank diff, | n1 | n2 | Z |
| 6h vs. 24h | 3,500 | 9,667 | -6,167 | 6 | 6 | 2,245 |
| 6h vs. 72h | 3,500 | 14,25 | -10,75 | 6 | 4 | 3,501 |
| 24h vs. 72h | 9,667 | 14,25 | -4,583 | 6 | 4 | 1,492 |

***Table S22*** *Statistical details for the physiological changes in the secretion levels of Fibronectin by IL1-β stimulation.*

| | | | | | | |
|---|---|---|---|---|---|---|
| Number of families | 1 | | | | | |
| Number of comparisons per family | 3 | | | | | |
| Alpha | 0,05 | | | | | |
| Uncorrected Dunn's test | Mean rank diff, | Significant? | Summary | Individual P Value | | |
| 6h vs. 24h | -9,000 | Yes | * | 0,0162 | A-B | |
| 6h vs. 72h | -18,00 | Yes | **** | <0,0001 | A-C | |
| 24h vs. 72h | -9,000 | Yes | * | 0,0162 | B-C | |
| Test details | Mean rank 1 | Mean rank 2 | Mean rank diff, | n1 | n2 | Z |
| 6h vs. 24h | 5,000 | 14,00 | -9,000 | 9 | 9 | 2,405 |
| 6h vs. 72h | 5,000 | 23,00 | -18,00 | 9 | 9 | 4,811 |
| 24h vs. 72h | 14,00 | 23,00 | -9,000 | 9 | 9 | 2,405 |

***Table S23*** *Statistical details for the physiological changes in the expression levels of Fibronectin by IL1-β stimulation.*

| | | | | | | |
|---|---|---|---|---|---|---|
| Number of families | 1 | | | | | |
| Number of comparisons per family | 3 | | | | | |
| Alpha | 0,05 | | | | | |
| Uncorrected Dunn's test | Mean rank diff, | Significant? | Summary | Individual P Value | | |
| 6h vs. 24h | 5,333 | No | ns | 0,0836 | A-B | |
| 6h vs. 72h | 11,67 | Yes | *** | 0,0002 | A-C | |
| 24h vs. 72h | 6,333 | Yes | * | 0,0399 | B-C | |
| Test details | Mean rank 1 | Mean rank 2 | Mean rank diff, | n1 | n2 | Z |
| 6h vs. 24h | 15,17 | 9,833 | 5,333 | 6 | 6 | 1,730 |
| 6h vs. 72h | 15,17 | 3,500 | 11,67 | 6 | 6 | 3,785 |
| 24h vs. 72h | 9,833 | 3,500 | 6,333 | 6 | 6 | 2,055 |

***Table S24*** *Statistical details for the physiological changes in the secretion levels of MMP10 by TNF-α stimulation.*

| | | | | | | |
|---|---|---|---|---|---|---|
| Number of families | 1 | | | | | |
| Number of comparisons per family | 3 | | | | | |
| Alpha | 0,05 | | | | | |
| Uncorrected Dunn's test | Mean rank diff, | Significant? | Summary | Individual P Value | | |
| 6h vs. 24h | -9,000 | Yes | * | 0,0162 | A-B | |
| 6h vs. 72h | -18,00 | Yes | **** | <0,0001 | A-C | |
| 24h vs. 72h | -9,000 | Yes | * | 0,0162 | B-C | |
| Test details | Mean rank 1 | Mean rank 2 | Mean rank diff, | n1 | n2 | Z |
| 6h vs. 24h | 5,000 | 14,00 | -9,000 | 9 | 9 | 2,405 |
| 6h vs. 72h | 5,000 | 23,00 | -18,00 | 9 | 9 | 4,811 |
| 24h vs. 72h | 14,00 | 23,00 | -9,000 | 9 | 9 | 2,405 |

***Table S25*** *Statistical details for the physiological changes in the expression levels of MMP10 by TNF-α stimulation.*

| | | | | | | |
|---|---|---|---|---|---|---|
| Number of families | 1 | | | | | |
| Number of comparisons per family | 3 | | | | | |
| Alpha | 0,05 | | | | | |
| Uncorrected Dunn's test | Mean rank diff, | Significant? | Summary | Individual P Value | | |
| 6h vs. 24h | 8,100 | Yes | ** | 0,0069 | A-B | |
| 6h vs. 72h | 7,000 | Yes | * | 0,0153 | A-C | |
| 24h vs. 72h | -1,100 | No | ns | 0,6846 | B-C | |
| Test details | Mean rank 1 | Mean rank 2 | Mean rank diff, | n1 | n2 | Z |
| 6h vs. 24h | 13,50 | 5,400 | 8,100 | 4 | 5 | 2,700 |
| 6h vs. 72h | 13,50 | 6,500 | 7,000 | 4 | 6 | 2,425 |
| 24h vs. 72h | 5,400 | 6,500 | -1,100 | 5 | 6 | 0,4062 |

***Table S26*** *Statistical details for the physiological changes in the secretion levels of Fibronectin by TNF-α stimulation.*

| | | | | | | |
|---|---|---|---|---|---|---|
| Number of families | 1 | | | | | |
| Number of comparisons per family | 3 | | | | | |
| Alpha | 0,05 | | | | | |
| Uncorrected Dunn's test | Mean rank diff, | Significant? | Summary | Individual P Value | | |
| 6h vs. 24h | -9,333 | Yes | * | 0,0126 | A-B | |
| 6h vs. 72h | -17,67 | Yes | **** | <0,0001 | A-C | |
| 24h vs. 72h | -8,333 | Yes | * | 0,0259 | B-C | |
| Test details | Mean rank 1 | Mean rank 2 | Mean rank diff, | n1 | n2 | Z |
| 6h vs. 24h | 5,000 | 14,33 | -9,333 | 9 | 9 | 2,494 |
| 6h vs. 72h | 5,000 | 22,67 | -17,67 | 9 | 9 | 4,722 |
| 24h vs. 72h | 14,33 | 22,67 | -8,333 | 9 | 9 | 2,227 |

***Table S27*** *Statistical details for the physiological changes in the expression levels of Fibronectin by TNF-α stimulation.*

| | | | | | | |
|---|---|---|---|---|---|---|
| Number of families | 1 | | | | | |
| Number of comparisons per family | 3 | | | | | |
| Alpha | 0,05 | | | | | |
| Uncorrected Dunn's test | Mean rank diff, | Significant? | Summary | Individual P Value | | |
| 6h vs. 24h | 3,300 | No | ns | 0,2713 | A-B | |
| 6h vs. 72h | 7,300 | Yes | ** | 0,0070 | A-C | |
| 24h vs. 72h | 4,000 | No | ns | 0,1659 | B-C | |
| Test details | Mean rank 1 | Mean rank 2 | Mean rank diff, | n1 | n2 | Z |
| 6h vs. 24h | 11,80 | 8,500 | 3,300 | 5 | 4 | 1,100 |
| 6h vs. 72h | 11,80 | 4,500 | 7,300 | 5 | 6 | 2,696 |
| 24h vs. 72h | 8,500 | 4,500 | 4,000 | 4 | 6 | 1,386 |

***Table S28*** *Statistical details for the physiological changes in the secretion levels of MCP-1 by TNF-α stimulation.*

| | | | | | | |
|---|---|---|---|---|---|---|
| Number of families | 1 | | | | | |
| Number of comparisons per family | 3 | | | | | |
| Alpha | 0,05 | | | | | |
| Uncorrected Dunn's test | Mean rank diff, | Significant? | Summary | Individual P Value | | |
| 6h vs. 24h | -9,000 | Yes | * | 0,0162 | A-B | |
| 6h vs. 72h | -18,00 | Yes | **** | <0,0001 | A-C | |
| 24h vs. 72h | -9,000 | Yes | * | 0,0162 | B-C | |
| Test details | Mean rank 1 | Mean rank 2 | Mean rank diff, | n1 | n2 | Z |
| 6h vs. 24h | 5,000 | 14,00 | -9,000 | 9 | 9 | 2,405 |
| 6h vs. 72h | 5,000 | 23,00 | -18,00 | 9 | 9 | 4,811 |
| 24h vs. 72h | 14,00 | 23,00 | -9,000 | 9 | 9 | 2,405 |

***Table S29*** *Statistical details for the physiological changes in the expression levels of MCP-1 by TNF-α stimulation.*

| | | | | | | |
|---|---|---|---|---|---|---|
| Number of families | 1 | | | | | |
| Number of comparisons per family | 3 | | | | | |
| Alpha | 0,05 | | | | | |
| Uncorrected Dunn's test | Mean rank diff, | Significant? | Summary | Individual P Value | | |
| 6h vs. 24h | 5,000 | No | ns | 0,0641 | A-B | |
| 6h vs. 72h | 9,000 | Yes | *** | 0,0009 | A-C | |
| 24h vs. 72h | 4,000 | No | ns | 0,1763 | B-C | |
| Test details | Mean rank 1 | Mean rank 2 | Mean rank diff, | n1 | n2 | Z |
| 6h vs. 24h | 11,50 | 6,500 | 5,000 | 6 | 4 | 1,852 |
| 6h vs. 72h | 11,50 | 2,500 | 9,000 | 6 | 4 | 3,333 |
| 24h vs. 72h | 6,500 | 2,500 | 4,000 | 4 | 4 | 1,352 |

## Supplementary References

1. Chen, Y.-W. *et al.* A three-dimensional model of human lung development and disease from pluripotent stem cells. *Nat Cell Biol* **19**, 542–549 (2017).

2. Fuchs, S. *et al.* Differentiation of human alveolar epithelial cells in primary culture: morphological characterization and synthesis of caveolin-1 and surfactant protein-C. *Cell Tissue Res* **311**, 31–45 (2003).

3. Lehmann, A. D. *et al.* An in vitro triple cell co-culture model with primary cells mimicking the human alveolar epithelial barrier. *Eur J Pharm Biopharm* **77**, 398–406 (2011).

4. Huh, D. *et al.* Reconstituting Organ-Level Lung Functions on a Chip. *Science* **328**, 1662–1668 (2010).

5. Hermanns, M. I. *et al.* Primary human coculture model of alveolo-capillary unit to study mechanisms of injury to peripheral lung. *Cell Tissue Res* **336**, 91–105 (2009).
